# Cancer associated mutations in Sec61γ alter the permeability of the ER translocase

**DOI:** 10.1371/journal.pgen.1009780

**Published:** 2021-08-30

**Authors:** Christopher M. Witham, Aleshanee L. Paxman, Lamprini Baklous, Robert F. L. Steuart, Benjamin L. Schulz, Carl J. Mousley

**Affiliations:** 1 Curtin Medical School, Curtin University, Bentley, Western Australia, Australia; 2 Curtin Health Innovation Research Institute, Curtin University, Bentley, Western Australia, Australia; 3 School of Chemistry and Molecular Biosciences, University of Queensland, Lucia, Queensland, Australia; HudsonAlpha Institute for Biotechnology, UNITED STATES

## Abstract

Translocation of secretory and integral membrane proteins across or into the ER membrane occurs via the Sec61 complex, a heterotrimeric protein complex possessing two essential sub-units, Sec61p/Sec61α and Sss1p/Sec61γ and the non-essential Sbh1p/Sec61β subunit. In addition to forming a protein conducting channel, the Sec61 complex maintains the ER permeability barrier, preventing flow of molecules and ions. Loss of Sec61 integrity is detrimental and implicated in the progression of disease. The Sss1p/Sec61γ C-terminus is juxtaposed to the key gating module of Sec61p/Sec61α and is important for gating the translocon. Inspection of the cancer genome database identifies six mutations in highly conserved amino acids of Sec61γ/Sss1p. We identify that five out of the six mutations identified affect gating of the ER translocon, albeit with varying strength. Together, we find that mutations in Sec61γ that arise in malignant cells result in altered translocon gating dynamics, this offers the potential for the translocon to represent a target in co-therapy for cancer treatment.

## Author summary

The first step in the biogenesis of secretory proteins is the targeting and translocation into the endoplasmic reticulum (ER). Secretory proteins enter the ER via a gated channel in the ER membrane called the translocon, a protein complex composed of Sec61p/Sec61α, Sbh1p/Sec61β and Sss1p/Sec61γ. As a protein conducting channel the translocon must be sealed in a regulated manner to prevent the free flow of ions and small molecules between the ER and cytosol. We have discovered that mutations in Sec61γ that arise in cancer affect this seal but not the ability of this protein complex to translocate secretory proteins into the ER. We hypothesise that altered translocon gating contributes to malignancy by influencing factors such as migration, autophagy and chemotherapy resistance.

## Introduction

The endoplasmic reticulum (ER) is the entry point into the secretory pathway [1,2]. To enter this organelle proteins are conducted through a channel known as the translocon [3]. Secretory proteins are marked by the presence of a signal sequence that comprises an N-terminal positively charged N-domain, a largely hydrophobic central H-domain, and a polar C-terminal cleavage site or C-domain [4,5]. The signal peptide instructs the targeting and subsequent translocation of a precursor through the ER translocase by one of two distinct mechanisms [6,7]. Proteins that possess a signal sequence of sufficient hydrophobicity are translocated co-translationally by a mechanism dependent on the signal recognition particle [8–10]. Translocation may also occur independent of the SRP [6] whereby a secretory protein is fully synthesised and then maintained in an unfolded, translocation competent state by cytosolic chaperones prior to post-translational translocation via the SEC complex in yeast [11,12].

The ER translocase is formed by the conserved Sec61 heterotrimeric complex [3]. In yeast the Sec61 complex is comprised of Sec61p, Sss1p and Sbh1p with the equivalent in mammalian organisms being Sec61α, Sec61γ and Sec61β respectively [7,13]. Within the complex, Sec61p forms the subunit through which proteins pass [14,15]. This essential subunit contains ten transmembrane domains (TMDs) [13] which create the two halves of Sec61p, TMDs 1–5 and TMDs 6–10 [13,16]. These halves are joined by an external loop between TMD 5 and TMD 6 (loop 5/6) [13]. A distinct hourglass shape results from the central constriction of the channel created by the pore ring, a series of hydrophobic residues that help to form a seal during translocation. While inactive, a plug formed by the first portion of TMD 2 (2a) resides within the pore ring [13]. This plug is partially displaced to allow for translocation to occur [13]. The two halves of Sec61p also form the lateral gate [13]. A hinge is formed between loop 5/6 which acts as an important regulator of translocon opening via facilitating exposure to the lipid bilayer of the ER membrane [13,17]. Ribosomal binding initiates partial opening of the lateral gate which is completed through the integration of the signal sequence between TMD 2 and TMD 7 [13,17].

Sss1p is an essential component of the translocon, acting to stabilise the conformation of the channel [18]. The amphipathic N-terminal helix and the TMD of Sss1p wraps around Sec61p on the surface and diagonally around TMDs 1, 5, 6 and 10 of Sec61p respectively, clamping the two halves of the structure. Sbh1p is only essential in higher eukaryotes. It contains one TM domain, the N-terminus of which makes contact with Sec61p [12,19,20]. The cytosolic domain of Sbh1p is largely unstructured and not visible in any of the available structures. As such, it is unknown to what extent this domain makes direct contact with Sec61, however, it is highly likely that it does so as this domain can be crosslinked to polypeptides as they translocate through the Sec61 complex [21].

The translocon must allow for passage of a protein while maintaining the permeability barrier between the cytosol and ER lumen. The ER environment facilitates luminal processes such as protein folding and appropriate cellular signalling. Disturbances to this system can result in ER stress which can lead to induction of recovery mechanisms including the unfolded protein response (UPR) [22,23]. During translocation there is opportunity for the movement of small molecules into and out of the ER [24,25]. Docking of the ribosome to the translocon during co-translational translocation initiates displacement of the plug usually residing within the inactive Sec61 complex [26]. As plug displacement occurs the translating protein is thread through the translocon, keeping the pore blocked and preventing the flow of molecules [24]. Upon the immediate completion of translation, the ribosome remains docked to the translocon in an idle state. [27,28]. Here, the Sec61 complex remains open and empty prior to the detachment of ribosomes [24,25]. At this stage small molecules can pass between the different cellular environments through the translocon [24,29]. The dissociation of the idle ribosome from the translocon causes a conformational shift within the Sec61 complex, closing the channel once again [30]. Work by Trueman *et al*. and Ponsero *et al*. demonstrated that mutations in Sec61p can destabilise the closed or open conformation of the translocon [24,29]. Destabilisation of the closed translocon increases the opportunity for molecules to pass into and out of the ER [24]. Conversely, destabilisation of the open Sec61 complex decreases movement of small molecules through the translocon [24].

The extreme C-terminus of Sss1p has been shown to be located adjacent to key amino acids in Sec61p that gate this channel and genetic analyses suggest a role for this region in gating the translocon [13]. Inspection of the cancer genome database identifies several mutations in highly conserved amino acids of Sss1p. We identify that five out of the six mutations identified could affect gating of the ER translocon, albeit with varying strength. Together, we find that mutations in Sec61γ that arise in malignant cells result in altered translocon gating dynamics, this offers the potential for the translocon to represent a target in co-therapy for cancer treatment.

## Results

### Sec61γ cancer associated mutations do not disrupt ER translocation

**Fig 1:**
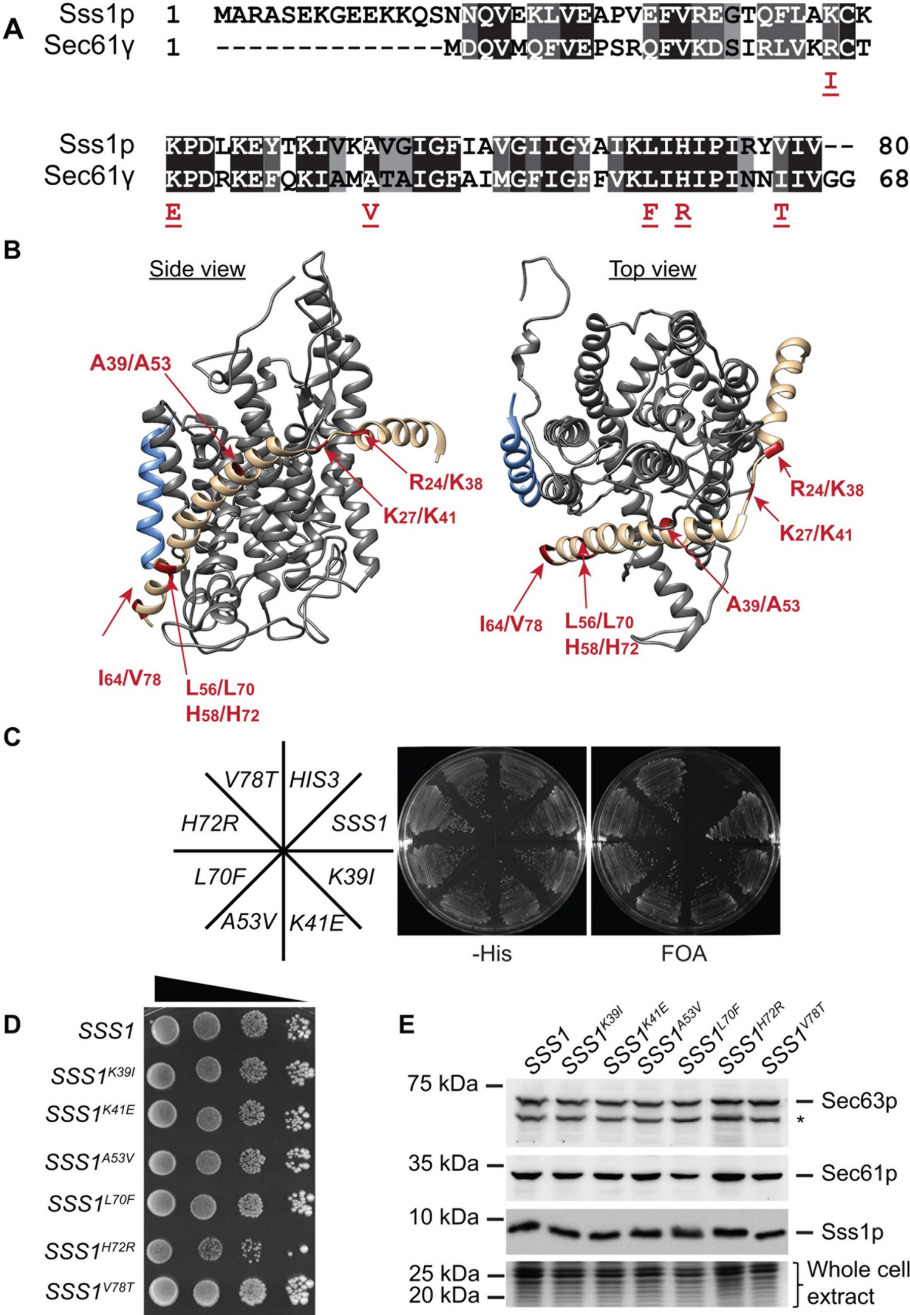
The Sss1p C-terminus is highly conserved. **(A)** The sequence of Sec61γ and Sss1p are aligned using clustal omega sequence alignment software and the position of cancer associated mutations indicated. **(B)** Ribbon diagram of the Sec61 complex crystal structure (4CG7.pdb) (32) was composed using Chimera software. Sec61α, Sec61β and Sec61γ, are coloured grey, blue and sand respectively. Cancer associated mutations are indicated in red. **(C)** BWY530 yeast transformed with either YCp *HIS3*, YCp *SSS1*, YCp *SSS1*^*K39I*^, YCp *SSS1*^*K41E*^, YCp *SSS1*^*A53V*^, YCp *SSS1*^*L70F*^, YCp *SSS1*^*H72R*^ or YCp *SSS1*^*V78T*^ were streaked onto–His selective medium and medium containing FOA and incubated at 30°C for 2 days. **(D)** Wildtype or cells expressing either *SSS1*^*K39I*^, *SSS1*^*K41E*^, *SSS1*^*A53V*^, *SSS1*^*L70F*^, *SSS1*^*H72R*^ or *SSS1*^*V78T*^ as the sole source of *SSS1* were spotted in a 10 fold dilution series and grown on YPD at 30°C for 3 days. **(E)** Cell extracts derived from wildtype cells or cells expressing either *SSS1*^*K39I*^, *SSS1*^*K41E*^, *SSS1*^*A53V*^, *SSS1*^*L70F*^, *SSS1*^*H72R*^ or *SSS1*^*V78T*^ were immunoblotted with anti-Sss1p, anti-Sec61p or anti-Sec63p antibodies. * identifies a proteolysed product of Sec63p.

Cancer genome databases provide a repository of naturally occurring mutations in genes that potentially impact the function of a protein they encode given their association with disease. We were interested to determine whether mutations in Sec61γ that arise in cancer alter gating dynamics of the Sec61 complex. Mining the COSMIC database identified 6 mutations in Sec61γ in residues that are highly conserved in eukaryotes (Fig 1A). The R24I mutation was identified in a patient with colorectal cancer, the K27E and I64T mutations were identified in patients with endometrial cancer, the A39V mutation in a patient with pancreatic cancer and the L56F and H58R mutations identified in patients with lung cancer. The equivalent mutations in Sss1p are K38I, K41E, A53V, L70F, H72R and V78T respectively and these are found throughout the protein (Fig 1B). Importantly, these mutations do not represent natural SEC61γ polymorphisms as none of these mutations are annotated in the genome aggregation database (gnomAD) that spans 125748 exome sequences and 15708 whole genome sequences from unrelated individuals [31]. We exploited our yeast model to test whether these mutations grossly alter Sss1p function. *SSS1* is an essential gene as *sss1Δ* cells are not viable. Therefore, we firstly tested if expression of these cancer associated variants could sustain cell viability via a plasmid shuffle assay. This method involves introduction of a plasmid containing a mutated copy of an essential gene into a strain carrying the wild-type gene on a *URA3* plasmid to complement the disruption of the chromosomal copy of the gene. This is followed by growth in the presence of 5-fluoroorotic acid (5-FOA) to prevent propagation of the *URA3* plasmid. 5-FOA resistant cells can only be isolated if the mutated copy of the gene retains sufficient essential activity. We transformed YCp *SSS1* and each mutant into BWY530 (*sss1Δ*::*KanMX4* FKp53) and tested for the ability of these strains to grow after counter-selecting for FKp53 on 5-FOA medium. Cells expressing plasmid derived copies of *SSS1* or any of the mutants produced viable colonies, whereas those transformed with vector alone could not, indicating that these mutations do not ablate function (Fig 1C and 1D).

Structurally Sss1p is composed almost exclusively of alpha helices. Importantly, the PSI-blast based secondary structure PREDiction (PSIPRED) program that uses artificial neural network machine learning algorithms to predict secondary structure [32] indicated that the cancer associated mutations did not alter Sss1p secondary structure (S1 Fig). Neither are key translocon subunits Sss1p and Sec61p and accessory proteins (e.g. Sec63p) affected in these mutants (Fig 1E). The biogenesis of DPAP B, which is translocated in an SRP dependent manner, prepro alpha factor, which is translocated post-translationally and Kar2p which can be translocated by both pathways were monitored to determine whether ER translocation is affected in these mutants. There was no obvious translocation defects in these mutants (S2A Fig). Finally, we investigated invertase secretion to determine if the secretory capacity of the cell is altered by our panel of cancer associated mutations. We observed no significant difference in the fraction of invertase that is secreted by the cell in each mutant when compared to wild type (S2B Fig). Therefore, the mutations in conserved residues in Sec61γ that arise in cancer do not perturb the essential translocation activity of the Sec61 complex nor do they alter the secretory capacity of the cell.

### The Sss1 H72R mutation affects translocon gating

**Fig 2:**
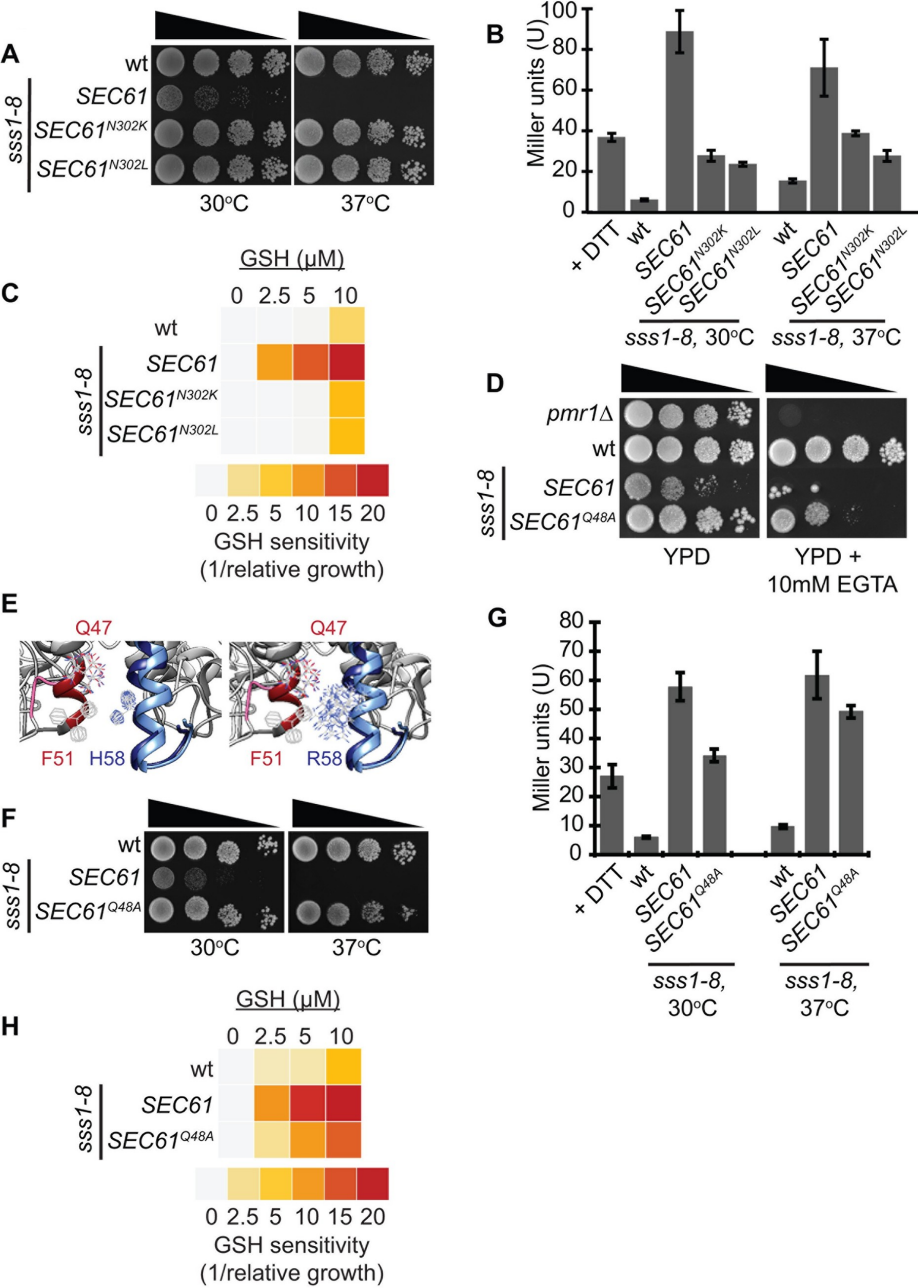
The *sss1*^*H72R*^ mutation disrupts ER homeostasis. **(A)** Wildtype or *sss1-8* yeast transformed with either YCp *SEC61*, YCp *SEC61*^*N302K*^ or YCp *SEC61*^*N302L*^ were spotted on YPD agar in a 10-fold dilution series and incubated at 30°C or 37°C for 2 days. **(B)** Wildtype or *sss1-8* yeast transformed with either YCp *SEC61*, YCp *SEC61*^*N302K*^ or YCp *SEC61*^*N302L*^ and with pJT30 (UPRE-LacZ) were grown in–Ura selective medium and β-Galactosidase activity determined. As a positive control wildtype cells were treated with 5mM DTT for 2 hours. **(C)** Wildtype or *sss1-8* yeast transformed with either YCp *SEC61*, YCp *SEC61*^*N302K*^ or YCp *SEC61*^*N302L*^ and with YEp *HGT1* were grown in–Ura selective medium with increasing concentrations of GSH. The relative growth of each strain determined and the GSH sensitivity (1/relative growth) presented. **(D)** Wildtype or *sss1-8 (sss1*^*H72R*^*)* yeast transformed with either YCp *SEC61* or YCp *SEC61*^*Q48A*^ were spotted on YPD agar or YPD agar containing 10mM EGTA in a 10-fold dilution series and incubated at 30°C for 3 days. **(E)** Ribbon diagram of the open (4CG5/7.pdb) and closed (4CG5/7.pdb) Sec61 complex crystal structure (34) was composed and overlayed using Chimera software. The position of Q47 and F51 in Sec61α relative to H58 in Sec61γ are indicated. **(F)** Wildtype or *sss1-8 (sss1*^*H72R*^*)* yeast transformed with either YCp *SEC61* or YCp *SEC61*^*Q48A*^ were spotted on YPD agar in a 10-fold dilution series and incubated at 30°C or 37°C for 2 days. **(G)** Wildtype or *sss1-8 (sss1*^*H72R*^*)* yeast transformed with either YCp *SEC61* or YCp *SEC61*^*Q48A*^ and with pJT30 (UPRE-LacZ) were grown in–Ura selective medium and β-Galactosidase activity determined. **(H)** Wildtype or *sss1-8 (sss1*^*H72R*^*)* yeast transformed with either YCp *SEC61* or YCp *SEC61*^*Q48A*^ and with YEp *HGT1* were grown in–Ura selective medium with increasing concentrations of GSH. The relative growth of each strain determined and the GSH sensitivity (1/relative growth) presented.

The H58 residue in Sec61γ is absolutely conserved in all homologues identified to date and corresponds to H72 in Sss1p [33,34]. Interestingly, the growth of cells expressing *sss1*^*H72R*^ is temperature sensitive as they are inviable at temperatures greater than 37°C (Fig 2A). However, the temperature sensitivity of *sss1*^*H72R*^ is not due to the stability of the key translocon subunits Sss1p and Sec61p and key accessory proteins Sec62p and Sec63p being affected in this mutant (see above; Fig 1D). Furthermore, the integrity of the translocon itself was not compromised in the *sss1*^*H72R*^ mutant. The Sss1p and Sec61p interaction can be stabilised by the crosslinking reagent disuccinimidyl suberate (DSS) [18,35]. We detected a DSS-dependent immunoreactive band of ≈ 46 kDa with both anti-Sss1p and anti-Sec61p specific antibodies in membranes isolated from wildtype or *sss1*^*H72R*^ cells treated with DSS, regardless of whether they were grown at 30°C or 37°C (S2C Fig).

Blue native polyacrylamide gel electrophoresis (BN-PAGE) has been used to resolve important translocation structures; specifically the Sec61 complex, the Sec63/71/72 subcomplex and the SEC’ and SEC complex [36]. We therefore used BN-PAGE to complement our cross-linking analysis. Microsomes isolated from wildtype cells that were solubilised with 2% digitonin yielded the 140 kDa Sec61 complex, the 350 kDa SEC’ complex and the 380 kDa SEC complex (S2D Fig). These complexes were all observed in microsomes isolated from *sss1-6*, *sss1-7* and *sss1-8* (*sss1*^*H72R*^) mutants (S2D Fig) further supporting the conclusion that the integrity of ER translocation complexes are not compromised in *sss1ts* mutants.

Given that activity of the Sec61 complex is essential for ER homeostasis, we tested if the unfolded protein response (UPR) was induced in *sss1*^*H72R*^ mutants. For this we used a lacZ reporter placed under transcriptional control of a yeast UPR enhancer (UPRE) [34]. WT cells were treated with the reducing agent dithiothreitol (DTT) to gauge a typical UPR response. UPR dependent Lac Z activity was significantly elevated in DTT treated cells compared to WT (Fig 2B). LacZ activity in *sss1*^*H72R*^ cells at 30°C and 37°C was up to 11-fold greater than that of WT. This confirms that the UPR is constitutively induced in *sss1*^*H72R*^ cells.

The sensitivities of *sss1*^*H72R*^ cell growth and degree of ER stress in the absence of an obvious ER translocation defect suggested to us that the *sss1*^*H72R*^ mutation may compromise the permeability of the translocon. We have found in a related study [34] that the *SEC61*^*N302L*^ mutation, a mutation in the lumenal lateral gate described by Gilmore and co-workers which destabilises the open conformation of the translocon [29], and *SEC61*^*N302K*^ suppresses the growth defects of other temperature sensitive *sss1* mutants in a dominant manner. Expression of either *SEC61*^*N302L*^ or *SEC61*^*N302K*^ from a centromeric plasmid also suppressed the temperature sensitive growth of *sss1*^*H72R*^ cells (Fig 2A) and reduced UPR induction in this mutant (Fig 2B). This suggests that translocon gating is defective in *sss1*^*H72R*^ mutants.

The Sec61 translocon has been shown to facilitate the diffusion of reduced glutathione (GSH) into the ER [24]. WT cells that overexpress Hgt1p, the plasma membrane high-affinity GSH transporter (↑HGT1 cells hereafter), amass high levels of GSH, when it is provided exogenously, that become cytotoxic due to a regulated response that results in hyper-oxidation of the ER lumen [24]. Using this system, we show that WT ↑Hgt1p cells easily tolerate up to 10 μM GSH (Fig 2C) Moreover, *sss1*^*H72R*^ ↑Hgt1p growth is extremely sensitive to GSH, as growth of these cells was severely perturbed by 2.5 μM GSH and 5 μM GSH, and completely arrested by 10 μM GSH (Fig 2C). Furthermore, the GSH hypersensitive growth defect of *sss1ts* mutants is not due to differential expression of *HGT1* (S3A and S3B Fig). Importantly, co-expression of *SEC61*^*N302L*^ or *SEC61*^*N302K*^ also suppressed the extreme sensitivity of *sss1*^*H72R*^ ↑Hgt1p growth in the presence of GSH (Fig 2C).

Farnesyl pyrophosphate (FPP) synthetase (Fpp1p) activity is Mn^2+^ dependent [37–39] and Fpp1p activity is elevated when cytoplasmic Mn^2+^ levels are raised, which results in increased squalene synthesis [37]. Squalene accumulation inhibits cell growth if it cannot be metabolised; such as when cells are treated with the squalene epoxidase inhibitor terbinafine [37]. We used this system to determine whether *sss1*^*H72R*^ cells possessed increased Fpp1p activity due to defective Mn^2+^ homeostasis. *sss1*^*H72R*^ cell growth was extremely sensitive to terbinafine as, unlike wildtype, 1 μg/ml terbinafine completely inhibited the growth of *sss1*^*H72R*^ mutants at 30°C and 34°C respectively (S3C Fig). Importantly, *sss1*^*H72R*^ cells are not hypersensitive to the 14α-sterol demethylase inhibitor miconazole (S3D Fig), indicating that the hypersensitivity of *sss1*^*H72R*^ cells to terbinafine is not due to general inhibition of the ergosterol biosynthetic pathway.

The cation content of the ER in yeast is controlled by both the Pmr1p and Spf1p/Cod1p P-type ATPases [40–42]. The growth of mutants that are defective in the storage of Ca^2+^ in secretory organelles, *pmr1Δ* and *spf1Δ* specifically, is hypersensitive to the presence of the Ca^2+^ chelator EGTA in the growth medium. Given this we hypothesised that the growth of *sss1* mutants defective in translocon gating would be hypersensitive to EGTA. Wildtype cell growth is resistant to up to 20 mM EGTA. However, *sss1*^*H72R*^ cells showed similar hypersensitivity to EGTA as *pmr1Δ* mutants (Fig 2D). Again, the EGTA hypersensitive growth defect of *sss1ts* mutants is not due to differential expression of *PMR1* (S3A and S3B Fig). Importantly the deleterious effects of EGTA on *sss1*^*H72R*^ growth are negated by the addition of exogenous Ca^2+^ to the growth medium (S3E Fig).

Taken together we conclude that the hypersentivities of *sss1*^*ts*^ growth to GSH, terbinafine and EGTA are due to the increased flux of GSH, Mn^2+^ and Ca^2+^, respectively, through the translocon in these mutants. Regarding the latter, however, we must acknowledge that we cannot rule out the possibility that the biogenesis of the Ca^2+^ pump is affected when Sss1p is mutated.

### Structural rationale for altered translocon gating in Sec61γ H58R mutant

When the structure of the translocation channel was first solved it was proposed that the most significant structural rearrangement that takes place upon opening of the translocon is the relocation of the plug domain, from within the central cavity of the closed channel, to a site adjacent to C-terminal portion of TM1 of SecY/Sec61α, the Sec61β TMD and the extreme C-terminus of SecE/Sec61γ in the open state [13]. However, structural analysis of the active mammalian translocon revealed it to only undergo subtle rearrangement as it transitions from an inactive to active state [43]. We note F51, located at the extreme C-terminus of Sec61α TM1, shifts and rotates towards the KLIHIPI peptide located near the extreme C-terminus of Sec61γ [43] (Fig 2E). This movement positions the sidechain of Q47 that flanks Sec61α TM1 close to that of Sec61γ H58 [43] (Fig 2E). We have modelled this structural feature in several of the most high resolution structures of the translocon, specifically 6ND1 (CryoEM structure of the Sec Complex from yeast) [44], 6R7Q (Structure of XBP1u-paused ribosome nascent chain complex with Sec61) [45], 6FTJ (Cryo-EM Structure of the Mammalian Oligosaccharyltransferase Bound to Sec61 and the Non-programmed 80S Ribosome) [46], 6Z3T (Structure of canine Sec61 inhibited by mycolactone) [47] and 6W6L (Cryo-EM structure of the human ribosome-TMCO1 translocon) [48], and have found these to be highly comparable (S4 Fig). Substitution of H58 with R would position the charged moiety of these side chains closer to one another, that may result in a strengthened interaction between these two residues that could stabilise the open conformation of the translocon (Fig 2E). We reasoned that disrupting this potential interaction would phenocopy the effect of mutations in the lumenal and lateral gate that destabilise the closed conformation of the translocon. Sec61α Q47 is well conserved with the corresponding residue being Sec61p Q48 in yeast. We tested whether *SEC61*^*Q48A*^ could suppress the temperature sensitivity of the *sss1*^*H72R*^ mutant in a dominant manner. Indeed, we found the suppressive effects of *SEC61*^*Q48A*^ to be indistinguishable from those of the *SEC61*^*N302L*^ mutant (Fig 2F). Furthermore, *SEC61*^*Q48A*^ could dominantly suppress all phenotypes associated with altered permeability of the ER translocase (Fig 2D, 2G and 2H).

### Other Sec61γ cancer associated mutations alter translocon gating

Mutations in *SEC61* that alter the gating dynamics of the translocon do not profoundly affect cell physiology under normal growth conditions. However, these mutations have been shown to dramatically affect the growth defects of *sss1* mutants that are defective in translocon gating; namely *sss1-6* and *sss1-7* (*sss1*^*P74A*, *175A*^ and *sss1*^*H72K*^ mutations respectively) [34]. Specifically, mutations in the lateral gate of Sec61p that destabilise the open conformation of the translocon, *SEC61*^*N302L*^ [29], completely suppress the ts growth defect of both *sss1-6* and *sss1-7* mutants [34] whereas a mutation that destabilises the closed conformation of the translocon, *SEC61*^*N302D*^ [29], further exacerbates the ts growth defect of *sss1-6* mutant, while the *sss1-7 SEC61*^*N302D*^ double mutant is inviable [34]. Therefore, we have a novel and elegant system that allows us to screen for mutations in components of the translocon and its associated proteins that destabilise either the open or the closed conformation of the translocon. That is mutations that destabilise the closed conformation of the translocon will exacerbate *sss1-6* temperature sensitivity, while, mutations that destabilise the open conformation of the translocon will suppress *sss1-6* and *sss1-7* growth defects.

**Fig 3:**
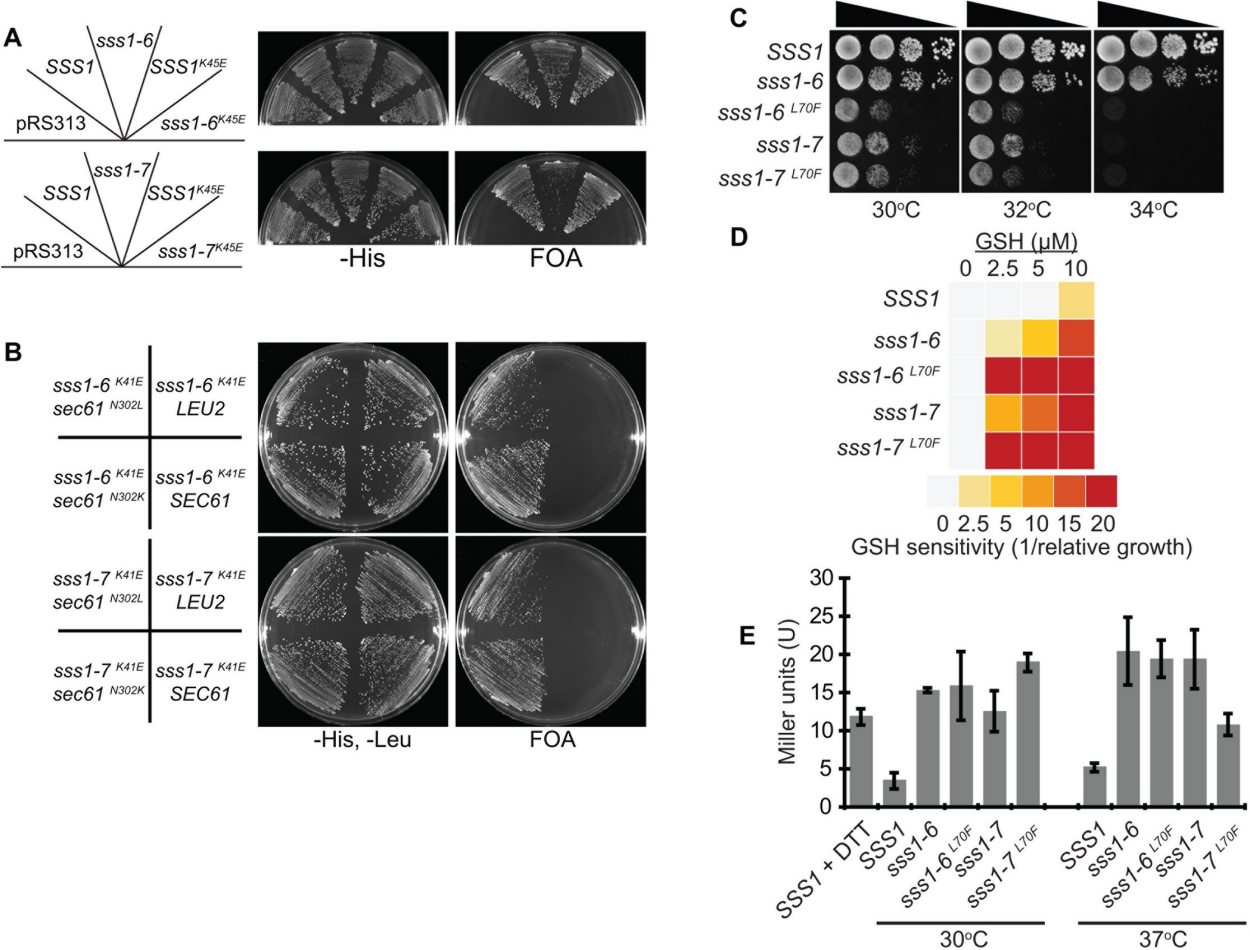
The K27E and L56F mutations destabilise the closed conformation of the translocon. **(A)** BWY530 yeast transformed with either YCp *HIS3*, YCp *SSS1*, YCp *SSS1*^*P74A*,*I75A*^, YCp *SSS1*^*K41E*, *P74A*,*I75A*^, YCp *SSS1*^*H72K*^, YCp *SSS1*^*K41E*,*H72K*^ were streaked onto–His selective medium and medium containing FOA and incubated at 30°C for 2 days. **(B)** BWY530 yeast transformed with YCp *SSS1*^*K41E*, *P74A*,*I75A*^ and YCp *LEU2*, YCp *SEC61*, YCp *SEC61*^*N302K*^ or YCp *SEC61*^*N302L*^ or YCp *SSS1*^*K41E*, *H72KA*^ and YCp *LEU2*, YCp *SEC61*, YCp *SEC61*^*N302K*^ or YCp *SEC61*^*N302L*^ were streaked onto–His selective medium and medium containing FOA and incubated at 30°C for 2 days. **(C)** Wildtype or cells expressing either *SSS1*^*P74A*,*I75A*^, *SSS1*^*L70F*, *P74A*,*I75A*^, *SSS1*^*H72K*^ or *SSS1*^*L70F*,*H72K*^ as the sole source of *SSS1* were spotted on YPD agar in a 10-fold dilution series and incubated at 30°C, 32°C or 34°C for 2 days. **(D)** Wildtype or cells expressing either *SSS1*^*P74A*,*I75A*^, *SSS1*^*L70F*, *P74A*,*I75A*^, *SSS1*^*H72K*^ or *SSS1*^*L70F*,*H72K*^ as the sole source of *SSS1* transformed with YEp *HGT1* were grown in–Ura selective medium with increasing concentrations of GSH. The relative growth of each strain determined and the GSH sensitivity (1/relative growth) presented. **(E)** Wildtype or cells expressing either *SSS1*^*P74A*,*I75A*^, *SSS1*^*L70F*, *P74A*,*I75A*^, *SSS1*^*H72K*^ or *SSS1*^*L70F*,*H72K*^ as the sole source of *SSS1* transformed with pJT30 (UPRE-LacZ) were grown in–Ura selective medium and β-Galactosidase activity determined. As a positive control wildtype cells were treated with 5mM DTT for 2 hours.

We combined each of the mutations described in Fig 1A with either *sss1*^*H72K*^ (*sss1-7*) or the less severe *sss1*^*P74A*, *I75A*^ (*sss1-6*) and investigated whether they suppressed or exacerbated *sss1-6* and *sss1-7* growth defects using the plasmid shuffle strain BWY530. We were unable to counter-select for FKp53 on FOA medium in cells expressing either *sss1-6* ^*K41E*^ or *sss1-7* ^*K41E*^ (Fig 3A). The simplest explanation for this is that the incorporation of K41E into either *sss1-6* and *sss1-7* results in a completely functionless Sss1p variant. An alternative explanation is that the magnitude of the translocon gating defect when the K41E mutation is combined with either *sss1-6* and *sss1-7* is such that cells are no longer viable. To discern between these two possibilities we reasoned that co-expression of the Sec61^N302L^p mutant, which destabilises the open conformation of the translocon, would restore viability to *sss1-6* ^*K41E*^ or *sss1-7* ^*K41E*^ if the latter scenario is correct. Indeed this was the case, as co-expression of *SEC61*^*N302L*^, but not *SEC61* alone, allowed either *sss1-6* ^*K41E*^ or *sss1-7* ^*K41E*^ to sustain cell viability when expressed as the sole copy of *SSS1* (Fig 3B).

A second mutation, L70F, was also found to exacerbate the growth defects of both *sss1-6*, and *sss1-7*. The growth of *sss1-6* ^*L70F*^ mutants at 30°C and 32°C was barely detectable after 2 days unlike *sss1-6* (Fig 3C). Furthermore, we discovered the GSH hypersensitive cell growth of *sss1-6* ^*L70F*^ and *sss1-7* ^*L70F*^ to be exacerbated relative to *sss1-6* and *sss1-7* respectively as the former mutants were unable to grow in the presence of 2.5 μM GSH whereas growth arrest of the latter mutants is observed at 10 μM GSH (Fig 3D). However, *sss1-6* ^*L70F*^ mutants, but not *sss1-6*, are inviable at the semi-permissive temperature of 34°C (Fig 3C). The UPR was induced to an equivalent extent in *sss1-6* ^*L70F*^ and *sss1-7* ^*L70F*^ relative to *sss1-6* and *sss1-7* (Fig 3E). This likely indicates that the extent with which the UPR is induced in these mutants has reached its maximum prior to the loss of cell viability.

**Fig 4:**
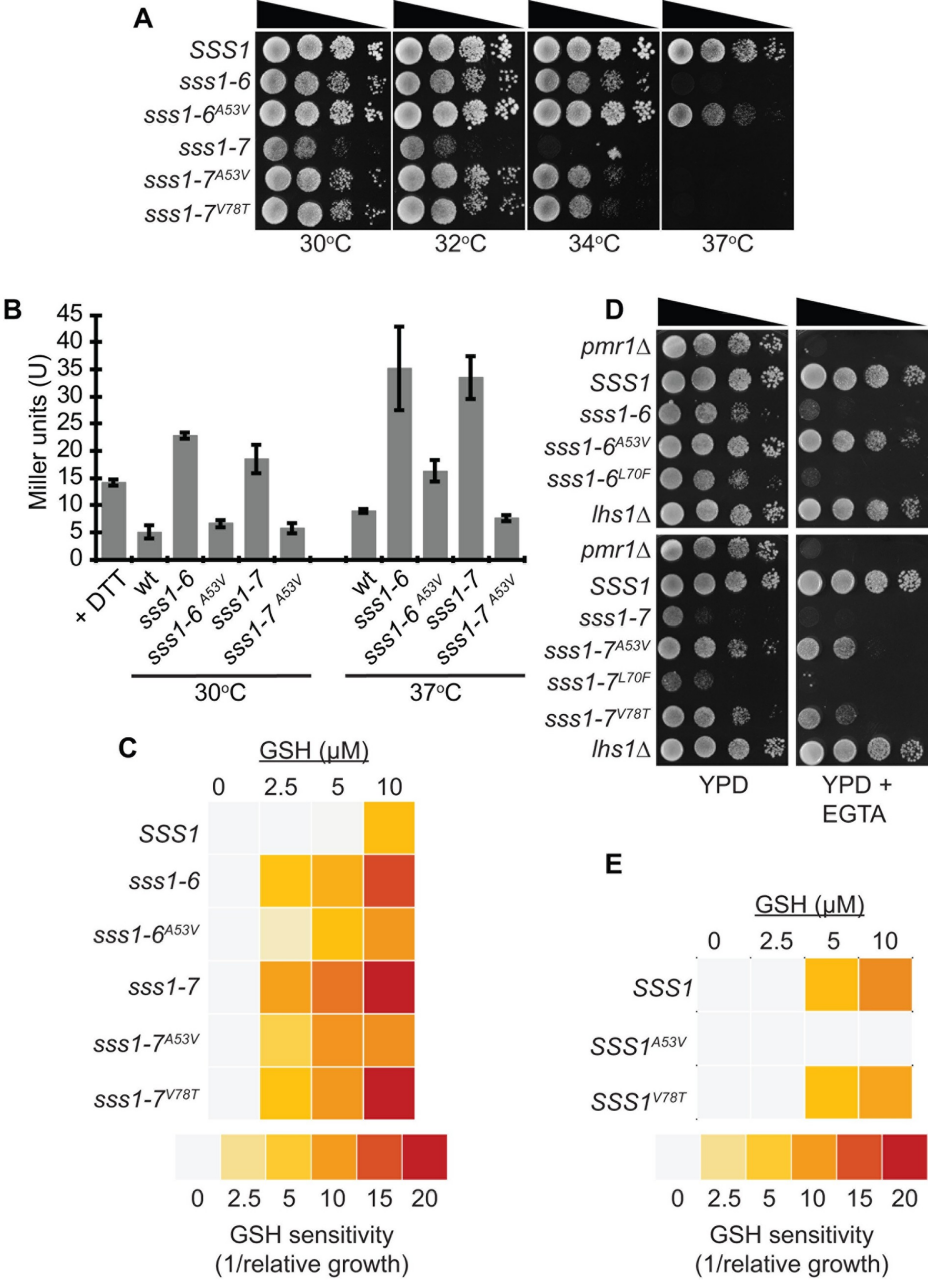
The A39V and I64T mutations destabilise the open conformation of the translocon. **(A)** Wildtype or cells expressing either *SSS1*^*P74A*,*I75A*^, *SSS1*^*A53V*, *P74A*,*I75A*^, *SSS1*^*H72K*^, *SSS1*^*A53V*, *H72K*^ or *SSS1*^*H72K*, *V78T*^ as the sole source of *SSS1* were spotted on YPD agar in a 10-fold dilution series and incubated at 30°C, 32°C, 34°C or 37°C for 2 days. **(B)** Wildtype or cells expressing either *SSS1*^*P74A*,*I75A*^, *SSS1*^*A53V*, *P74A*,*I75A*^, *SSS1*^*H72K*^, *SSS1*^*A53V*, *H72K*^ or *SSS1*^*H72K*, *V78T*^ as the sole source of *SSS1* transformed with pJT30 (UPRE-LacZ) were grown in–Ura selective medium and β-Galactosidase activity determined. As a positive control wildtype cells were treated with 5mM DTT for 2 hours. **(C)** Wildtype or cells expressing either *SSS1*^*P74A*,*I75A*^, *SSS1*^*A53V*, *P74A*,*I75A*^, *SSS1*^*H72K*^, *SSS1*^*A53V*, *H72K*^ or *SSS1*^*H72K*, *V78T*^ as the sole source of *SSS1* transformed with YEp *HGT1* were grown in–Ura selective medium with increasing concentrations of GSH. The relative growth of each strain determined and the GSH sensitivity (1/relative growth) presented. **(D)** Wildtype or cells expressing either *SSS1*^*P74A*,*I75A*^, *SSS1*^*A53V*, *P74A*,*I75A*^, *SSS1*^*H72K*^, *SSS1*^*A53V*, *H72K*^ or *SSS1*^*H72K*, *V78T*^ as the sole source of *SSS1* were spotted on YPD agar or YPD agar containing 5 mM (*sss1-7* derivatives) or 10 mM (*sss1-6* derivatives) EGTA in a 10-fold dilution series and incubated at 30°C for 3 days. **(E)** Wildtype or cells expressing either *SSS1*^*A53V*^ or *SSS1* ^*V78T*^ as the sole source of *SSS1* transformed with YEp *HGT1* were grown in–Ura selective medium with increasing concentrations of GSH. The relative growth of each strain determined and the GSH sensitivity (1/relative growth) presented.

In contrast to K41E and L70F, we find that two mutations, A53V and V78T, have suppressive effects on either both *sss1* mutants (A53V) or *sss1-7* only (V78T). *sss1-6* ^*A53V*^ could grow at 37°C whereas *sss1-7* ^*A53V*^ could grow at 34°C unlike *sss1-6* and *sss1-7* respectively (Fig 4A) and the extent with which the UPR was induced was less in both *sss1-6* ^*A53V*^ and *sss1-7* ^*A53V*^ (Fig 4B). The *sss1-7* ^*V78T*^ mutant could also grow at 34°C (Fig 4A) and the level to which the UPR was induced in *sss1-7* ^*V78T*^ was less than that observed for *sss1-7* (Fig 4B). We speculate that the P75A mutation in *sss1-6* alters the structure of the C-terminus such that the suppressive effects of the V78T mutation are negated in this mutant. The suppressive effects of both the A53V and V78T mutations also extended to overturn phenotypes associated with altered ER permeability. The A53V mutation was able to suppress the hypersensitivity of both *sss1-6* and *sss1-7* mutants to GSH (Fig 4C) and the V78T mutation did so for *sss1-7* (Fig 4C). Furthermore, A53V and V78T, have suppressive effects on either both *sss1* mutants (A53V) or *sss1-7* only (V78T) on EGTA hypersensitivity (Fig 4D) and terbinafine hypersensitivity (S5A and S5B Fig), albeit with varying strength.

Given the suppressive effects of the V78T and A53V mutations, described above, we were keen to determine whether these mutations alone were more resistant than *SSS1* to the cytotoxic effects of exogenous GSH. *SSS1*^*V78T*^ cells were only found to be fractionally more resistant to GSH than wt cells, however, *SSS1*^*A53V*^ cells were significantly more resistant to the deleterious effects of exogenous GSH (Fig 4E).

## Discussion

The Sec61 translocon facilitates the translocation of nascent proteins into the ER while maintaining the barrier between the two distinct environments of the ER lumen and cytosol. Additionally, the translocon’s capability to allow the controlled flux of essential metabolites across the ER membrane is vital to maintaining these functional environments as well as coordinating cellular processes that are regulated by small molecules. The dynamic nature of the translocon is fundamental in this channel’s ability to participate in these distinct functions; and while other ER channels have been described with roles in cancer and its progression [49,50], the involvement of dysregulated translocon dynamics had yet to be reported. Herein, we have demonstrated a mechanism by which mutations in the essential translocon subunit, Sec61γ /Sss1p, influence translocon gating. Furthermore, we show that cancer associated mutations of Sec61γ /Sss1p present with an ability to influence the stability of the translocon’s conformational states, stabilising either the closed or open state.

### A possible mechanism for *sss1*^*H72R*^ in disrupting gating dynamics

An intricate network of molecular interactions regulates the opening and closing of the translocon. N302 contributes to this network within the lateral and luminal gate that functions in setting the hydrophobicity threshold of the translocon [29]. The incorporation of a signal sequence into the channel disrupts this network under normal conditions, facilitating the transition to an open state [29]. Increasing the hydrophobicity of key residues (i.e N302L) complements non-polar interactions at the luminal and lateral gate which destabilises the open conformation [29]. Seeking a mechanism by which *sss1*^*H72R*^ disrupts gating dynamics, we inspected the structure of the active mammalian translocon, which revealed that upon translocon opening the side chain of the Sec61α TM1 residue Q47 is juxtaposed to H58 of Sec61γ. The substitution of H58 with R positions the charged moiety of these side chains within 2.1 Å of Q47 which may facilitate the formation of a strong non-covalent interaction between these two residues. This may affect the ability of the translocon to respond appropriately to signals for closure, therefore disrupting gating dynamics via stabilising the open state.

### Disrupting translocon dynamics: an outcome in cancer related mutations

Data presented in the human protein atlas suggests SEC61γ to be a prognostic marker for renal and liver cancer whereby high expression is shown to be unfavourable in both cancers. In light of this we were interested to see if there existed cancer associated mutations in SEC61γ that had any effect on function. Search of the cancer genome database revealed there to be six mutations in conserved residues. Significantly, these mutations are not just natural polymorphisms as they are documented in the genome aggregation database. Rather, they represent *bona fide* mutations that have arisen in patients with cancer. Utilising our *sss1* mutants (*sss1-6* and *sss1-7*) we have developed a system for the assessment of perturbations in translocon gating dynamics. These mutants destabilise the closed conformation of the channel, therefore the introduction of a further mutation to these mutants can have one of three possible outcomes: no effect on translocon gating, suppression which indicates an ability to destabilise the open and exacerbation which is an outcome of destabilising the closed further. Initially there was no apparent phenotype observed in the cancer associated mutations of Sss1p with the exception of *sss1*^*H72R*^. However, upon introduction into our system we found 4 (K41E, A53V, L70F, V78T) out of the remaining 5 also demonstrated an ability to influence translocon gating dynamics. It is important to indicate that the mutations in Sec61γ listed in the COSMIC database are alone, unlikely to be causative, driver mutations. However, these mutations legitimately alter the permeability of the ER translocase therefore we consider these mutations to be advantageous to cell fitness at a later stage of disease, such as when chemotherapy is administered or when a tumour metastasises. A subset of single nucleotide variants, proposed as passengers in cancer, has been shown to influence tumour progression [51].

Cellular compartmentalization has served as a significant advantage for eukaryotic cells by facilitating specialization of numerous cellular processes [52]. The ER has a distinct environment that promotes the processing and maturation of proteins [53,54]. Ca^2+^ contributes to establishing this environment and is present in abundance, particularly in mammalian cells where the ER is the major store for this ion [55] where it is utilised by molecular chaperones to facilitate protein folding [56]. In addition to its role at the ER, Ca^2+^ also regulates cell signalling, metabolism, autophagy and apoptosis; i.e. pathways manipulated in cancer [57]. Interestingly, the disparate effects of these cancer associated SEC61γ mutations appears to reflect the diverse way in which Ca^2+^ signalling affects cancer. The increasing energy demand of certain cancers can lead to the sustained transfer of Ca^2+^ from the ER to the mitochondria [57] which serves to fuel mitochondrial bioenergetics resulting in the production of ATP. Interestingly, in pancreatic ductal adenocarcinoma (PDAC) IP3Rs and STIM1 are reorganised to the leading edge of migrating cells [58]. Inhibition of IP3Rs and SOCE repressed migration demonstrating the importance of these mechanisms in this process [58]. As migration is energy demanding, the redistribution of these mechanisms likely represents the increasing demand for their role in enhancing mitochondrial bioenergetics. In the same vein, some cancers have demonstrated an ability to preferentially express certain isoforms of IP3R, i.e. upregulation of IP3R3 involved in calcium transport at MAMs [59]. These findings establish a need for some cancers to hoard calcium at the ER in order to sustain energy production. The A39V and I64T mutations, identified in PDAC and endometroid carcinoma respectively, might represent a contributing factor in this process. These mutations stabilise the closed conformation of the translocon, which could serve to reduce ion leakage.

The literature reveals some lung cancer cell lines possess reduced ER Ca^2+^ levels. Down regulating the import of Ca^2+^ levels makes the ER vulnerable to calcium leak. Increased cytosolic Ca^2+^ can induce autophagic flux that acts to compensate for metabolic stress via supplying nucleotides for cellular processes such as the TCA cycle and DNA repair [60]. These lung cancer cell lines show chemoresistance likely representing diminished ER to mitochondria Ca^2+^ transfer which is critical for induction of cell death [61,62]. Furthermore, cancerous cells develop an increased demand for protein and lipid biogenesis and therefore must adapt to and increasingly nutrient deprived environment. Uncontrolled ion movement from the ER can result in cellular stress and induce the UPR [22,63,64]. While prolonged cellular stress would typically induce apoptosis, some malignant cells can bypass apoptosis and utilise UPR to increase the protein folding capacity of the ER which can increase metastasis and chemotherapy resistance [65]. Collectively these finding demonstrate that depletion of ER Ca^2+^ stores can prove beneficial to the progression of certain cancers. Sss1p cancer mutations found to destabilise the closed / stabilise the open translocon include L56F and H58R, mutations isolated from lung squamous cell carcinoma as well as K27E of endometroid carcinoma origin. We propose that mutations that impose such an effect on the translocon could perpetuate Ca^2+^ flux, contributing to cancer outcomes.

This work has served as a proof of concept for our system in determining influences on translocon gating dynamics. This system could be utilised for future studies investigating components/regions that have yet to be characterised to such a role. Here, this system has set a precedent as a useful tool in identifying potential manipulations of translocon gating dynamics which may act in benefit of carcinogenesis and tumour progression. To our knowledge this is the first study to identify mutations in the SEC61γ gene that affect ER permeability to be associated with pathology. Given that pathologies have been found to be associated with genes encoding for translocon components (SEC61α1 and SEC61β) as well as translocon associated proteins (SEC62 and SEC63) we anticipate that several channelopathies that alter the permeability of the ER membrane may be associated with mutations in Sec61γ. If so we have a novel and elegant system in place that allows screening for such mutations.

## Materials and methods

### Yeast strains

Yeast strains (S1 Table) were grown in YP medium (2% peptone, 1% yeast extract) in the presence of 2% glucose (YPD). Growth was predominately performed at 30°C except where defined otherwise for the purposes of TS growth analysis which involved spotting onto media at a 10-fold dilution series. Minimal medium (0.67% yeast nitrogen base; YNB) with the addition of 2% glucose and appropriate supplements (20 μg/ml) was utilised for nutrient selection. 2% (w/v) agar was additionally added for solid media. Minimal media was prepared similarly yet with the addition of 1 g/L 5-fluoroorotic acid (5-FOA) and 100 μg/ml uracil to achieve counter selection of *URA3* plasmids. 1 μg/ml terbinafine or DMSO was added to YPD agar where indicated.

### Plasmid construction–site directed mutagenesis

Site directed mutagenesis was performed according to Q5 Site Directed Mutagenesis Protocol (NEB), the plasmids and oligonucleotides used are listed in S2 and S3 Tables respectively. The plasmid pJKB2 was used as template to introduce the desired mutations into *SSS1*.

### Glutathione sensitive growth assay

Yeast strains containing the YEp *HGT1* were cultured at 30°C to mid-logarithmic phase. Sub-cultures at 0.01 OD_600nm_ in SC medium were produced omitting uracil and with the addition of 0–10 μM of L-reduced glutathione. Growth was followed and recorded at several key time points. Three independent biological replicates and at least two technical replicates were performed. These results were averaged with each concentration compared as a factor of the 0 μM result.

### β-Galactosidase assays

β-Galactosidase assays were performed according to Tyson and Stirling, 2000 (66). Specifically, overnight yeast cultures were diluted to 0.2 OD_600nm_ and left to recover for 4 hrs at 30°C. Following a 2 hr temperature shift cells were harvested and resuspended in 2 ml of Z buffer (60 mM Na_2_HPO_4_, 40 mM NaH_2_PO_4_, 10 mM KCl, 10 mM MgSO_4_, 50 mM 2-mercaptoethanol, pH 7.0). Reaction mixes were made from 0.8 ml of cell suspension, 50 μl of 0.1% (w/v) SDS and 100 μl of CHCl_3_ and placed at 30°C for 30 mins to achieve cellular permeabilization. 160 μl of o-nitrophenylgalactopyranoside (4 mg/ml stock) was added to initiate the reaction for a 20 min duration. The addition of 400 μl of 1 M Na_2_CO_3_, pH 9.0 acted to halt the reaction. The OD_420nm_ was measured, and LacZ activity (U) was calculated by multiplying OD_420nm_/OD_600nm_ by 1000. Three independent biological replicates and at least two technical replicates were performed.

### Cell lysate preparation and immunoblotting

Yeast cells were grown to mid-logarithmic phase where 10 OD_600nm_ of cells were isolated for generation of crude cell lysates. Pelleted cells were resuspended in sample buffer with 0.5 mm glass beads. Samples were heated for 10mins at 65°C and disrupted via FastPrep-24 (6.0m/sec for 40 sec). Samples were placed back on heat until use or stored. Samples were run via SDS page and subsequently transferred to PVDF via a semi-dry transfer apparatus. Immunoblotting Antibodies used are listed in S4 Table.

## Supporting information

S1 Fig The primary sequence of Sss1p, Sss1K38Ip, Sss1K43Ep, Sss1A53Vp, Sss1L70Fp, Sss1H72Rp and Sss1V78Tp was analysed by PSIPRED 4.0 software (31). (TIF)

S2 Fig **(A)** Cell extracts derived from cells expressing either *SSS1* with or without tunicamycin (tm), *SSS1*^*K39I*^, *SSS1*^*K41E*^, *SSS1*^*A53V*^, *SSS1*^*L70F*^, *SSS1*^*H72R*^ or *SSS1*^*V78T*^ were immunoblotted with anti-DPAP B, anti-Kar2p and anti-ppαf antibodies. **(B)** Invertase secretion was determined in cells expressing either *SSS1*, *SSS1*^*K39I*^, *SSS1*^*K41E*^, *SSS1*^*A53V*^, *SSS1*^*L70F*^, *SSS1*^*H72R*^ or *SSS1*^*V78T*^ **(C)** Membranes derived from wildtype or *sss1*^*H72R*^ yeast incubated with and without 1 mM DSS were immunoblotted with anti-Sss1p and anti-Sec61p antibodies. **(D)** Two A_260nm_ units of microsomes prepared from wild type, *sss1-6 (sss1*^*P74A*, *I75A*^*)*, *sss1-7 (sss1*^*H72K*^*)* and *sss1-8 (sss1*^*H72R*^*)* were resolved by 6–16% BN-PAGE and analysed by Western blotting for Sec63p (upper panel) and Sec61p (lower panel).
(TIF)

S3 Fig **(A)***HGT1* and *PMR1* expression was determined by RT-PCR on cDNA derived from mRNA isolated from wildtype, *sss1-6*, *sss1-7* and *sss1-8* yeast harbouring YEp *HGT1*. **(B)** Expression of *HGT1* and *PMR1* relative to *ACT1* in wildtype, *sss1-6*, *sss1-7* and *sss1-8* yeast was detetermined. The histogram shows the average of at least 6 experiments. **(C)** Wildtype, *sss1-6*, *sss1-7* and *sss1-8* yeast were spotted on YPD agar or YPD agar containing 1 μg/mL terbinafine in a 10-fold dilution series and incubated at 30°C for 3 days. **(D)** Wildtype, *sss1-6*, *sss1-7* and *sss1-8* yeast were spotted on YPD agar or YPD agar containing 50 ng/mL miconazole in a 10-fold dilution series and incubated at 30°C for 3 days. **(E)** The relative growth of wild type, *pmr1Δ* and *sss1-8* cells, grown with and without CaCl_2_, was determined when grown with either 0, 0.25 mM, 0.5 mM, 1 mM or 2.5 mM EGTA.
(TIF)

S4 Fig Ribbon diagram of the Sec61 complex from five recent high resolution crystal structures; 6ND1 (43), 6R7Q (44), 6FTJ (45), 6Z3T (46) and 6W6L (47), are visualised using Chimera software. The position of Q47 in Sec61α relative to H58 in Sec61γ are indicated.
(TIF)

S5 Fig **(A)** Wildtype or cells expressing either *SSS1*^*P74A*,*I75A*^, *SSS1*^*L70F*, *P74A*,*I75A*^, *SSS1*^*H72K*^ or *SSS1*^*L70F*,*H72K*^ as the sole source of *SSS1* were spotted on YPD agar or YPD agar containing 1 μg/mL terbinafine in a 10-fold dilution series and incubated at 30°C, 32°C or 34°C for 2 days. **(B)** Wildtype or cells expressing either *SSS1*^*P74A*,*I75A*^, *SSS1*^*A53V*, *P74A*,*I75A*^, *SSS1*^*H72K*^, *SSS1*^*A53V*, *H72K*^ or *SSS1*^*H72K*, *V78T*^ as the sole source of *SSS1* were spotted on YPD agar or YPD agar containing 1 μg/mL terbinafine in a 10-fold dilution series and incubated at 30°C or 32°C for 2 days.
(TIF)

S1 Table Yeast strains used in this study. (PDF)

S2 Table Plasmids used in this study. (PDF)

S3 Table Oligonucleotides used in this study. (PDF)

S4 Table Antibodies used in this study. (PDF)

S1 Methods Supplemental materials, methods and references. (PDF)
