# Supplementary figures and images for: Cancer associated mutations in Sec61γ alter the permeability of the ER translocase
Source: PLoS Genet. 2021 Aug 30;17(8):e1009780. doi: 10.1371/journal.pgen.1009780 (PMC8439465; doi:10.1371/journal.pgen.1009780)

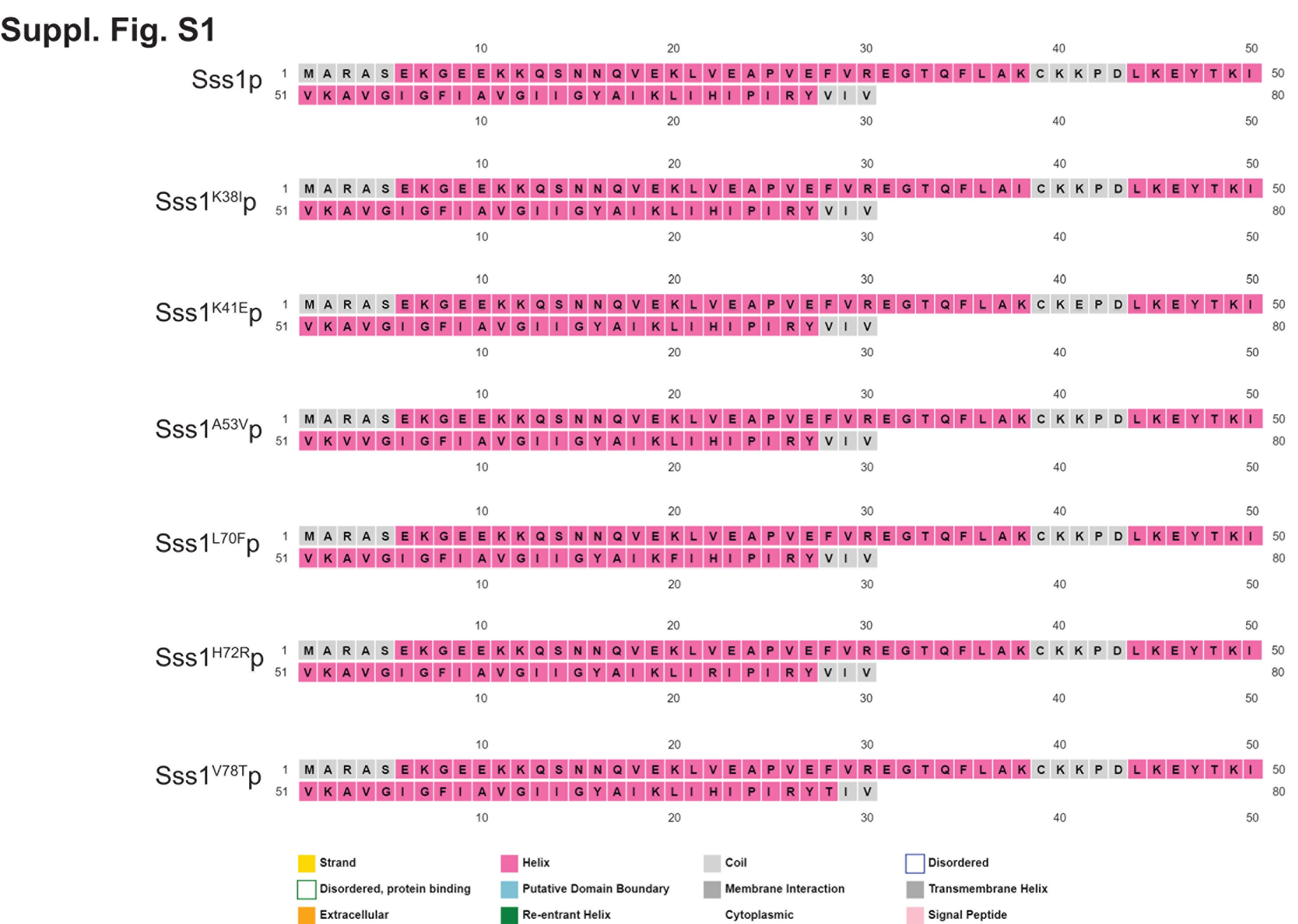

Supplement: S1 Fig — (TIF) [file pgen.1009780.s001.tif]

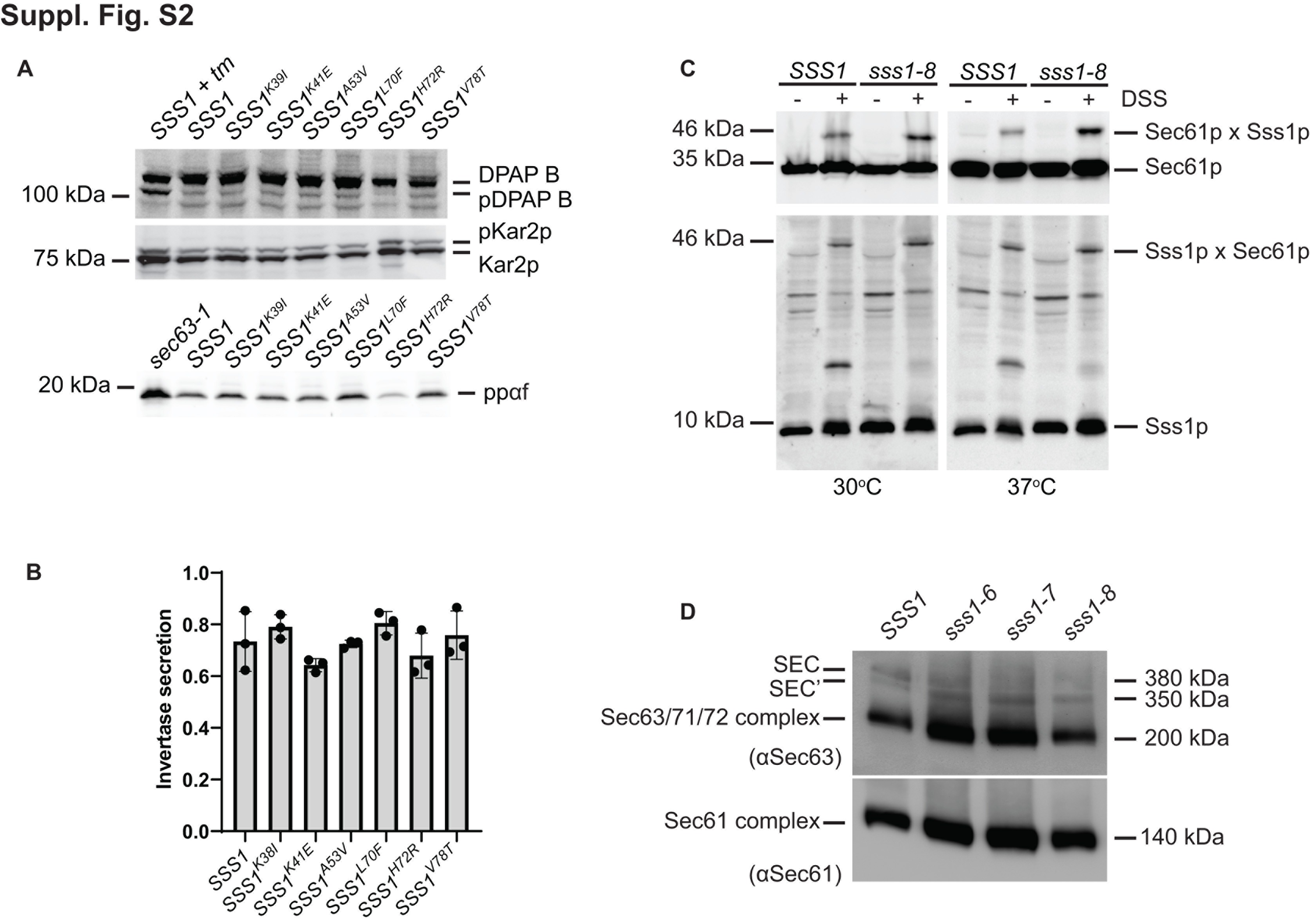

Supplement: S2 Fig — (A) Cell extracts derived from cells expressing either SSS1 with or without tunicamycin (tm), SSS1K39I, SSS1K41E, SSS1A53V, SSS1L70F, SSS1H72R or SSS1V78T were immunoblotted with anti-DPAP B, anti-Kar2p and anti-ppαf antibodies. (B) Invertase secretion was determined in cells expressing either SSS1, SSS1K39I, SSS1K41E, SSS1A53V, SSS1L70F, SSS1H72R or SSS1V78T (C) Membranes derived from wildtype or sss1H72R yeast incubated with and without 1 mM DSS were immunoblotted with anti-Sss1p and anti-Sec61p antibodies. (D) Two A260nm units of microsomes prepared from wild type, sss1-6 (sss1P74A, I75A), sss1-7 (sss1H72K) and sss1-8 (sss1H72R) were resolved by 6–16% BN-PAGE and analysed by Western blotting for Sec63p (upper panel) and Sec61p (lower panel). (TIF) [file pgen.1009780.s002.tif]

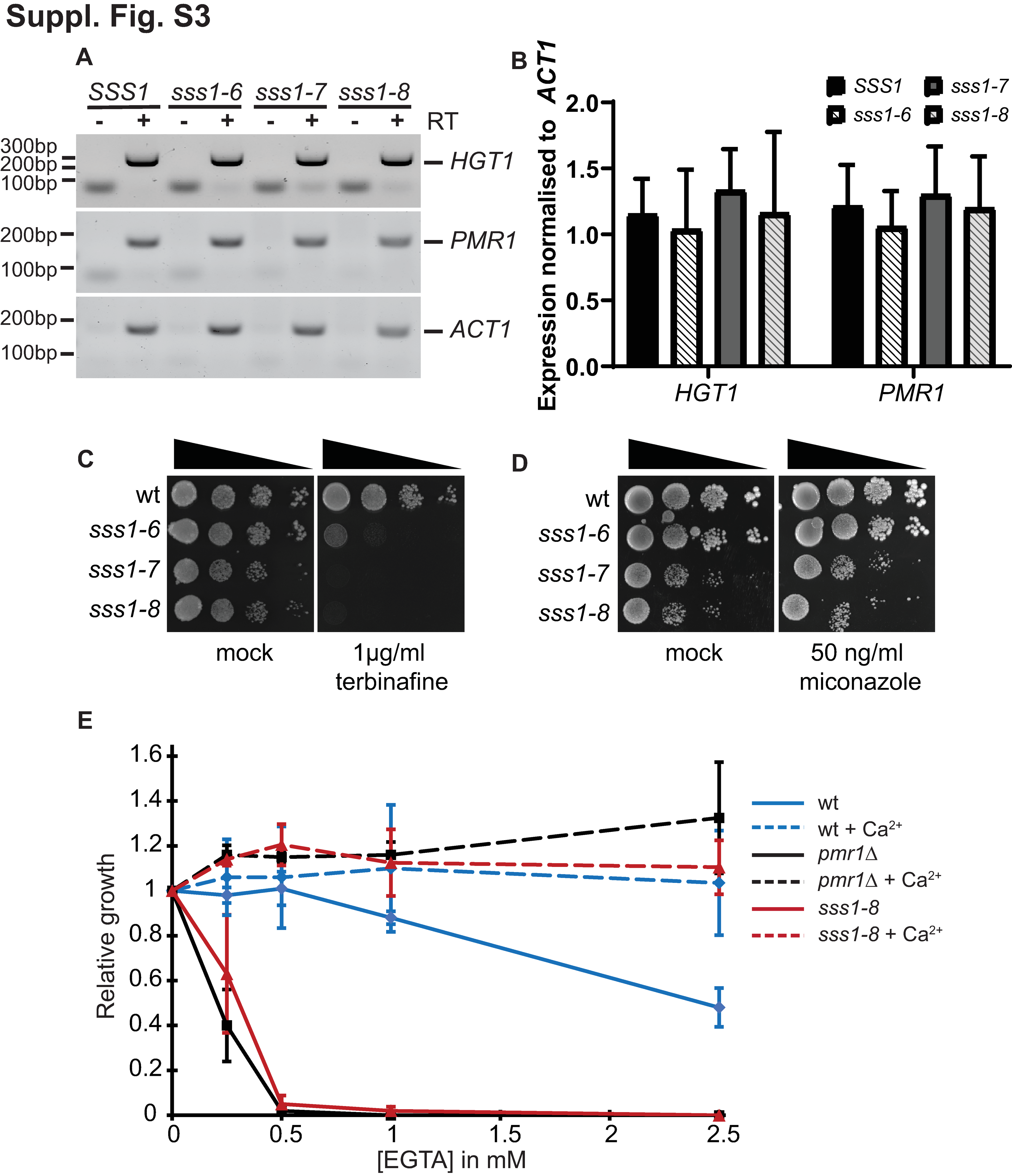

Supplement: S3 Fig — (A)HGT1 and PMR1 expression was determined by RT-PCR on cDNA derived from mRNA isolated from wildtype, sss1-6, sss1-7 and sss1-8 yeast harbouring YEp HGT1. (B) Expression of HGT1 and PMR1 relative to ACT1 in wildtype, sss1-6, sss1-7 and sss1-8 yeast was detetermined. The histogram shows the average of at least 6 experiments. (C) Wildtype, sss1-6, sss1-7 and sss1-8 yeast were spotted on YPD agar or YPD agar containing 1 μg/mL terbinafine in a 10-fold dilution series and incubated at 30°C for 3 days. (D) Wildtype, sss1-6, sss1-7 and sss1-8 yeast were spotted on YPD agar or YPD agar containing 50 ng/mL miconazole in a 10-fold dilution series and incubated at 30°C for 3 days. (E) The relative growth of wild type, pmr1Δ and sss1-8 cells, grown with and without CaCl2, was determined when grown with either 0, 0.25 mM, 0.5 mM, 1 mM or 2.5 mM EGTA. (TIF) [file pgen.1009780.s003.tif]

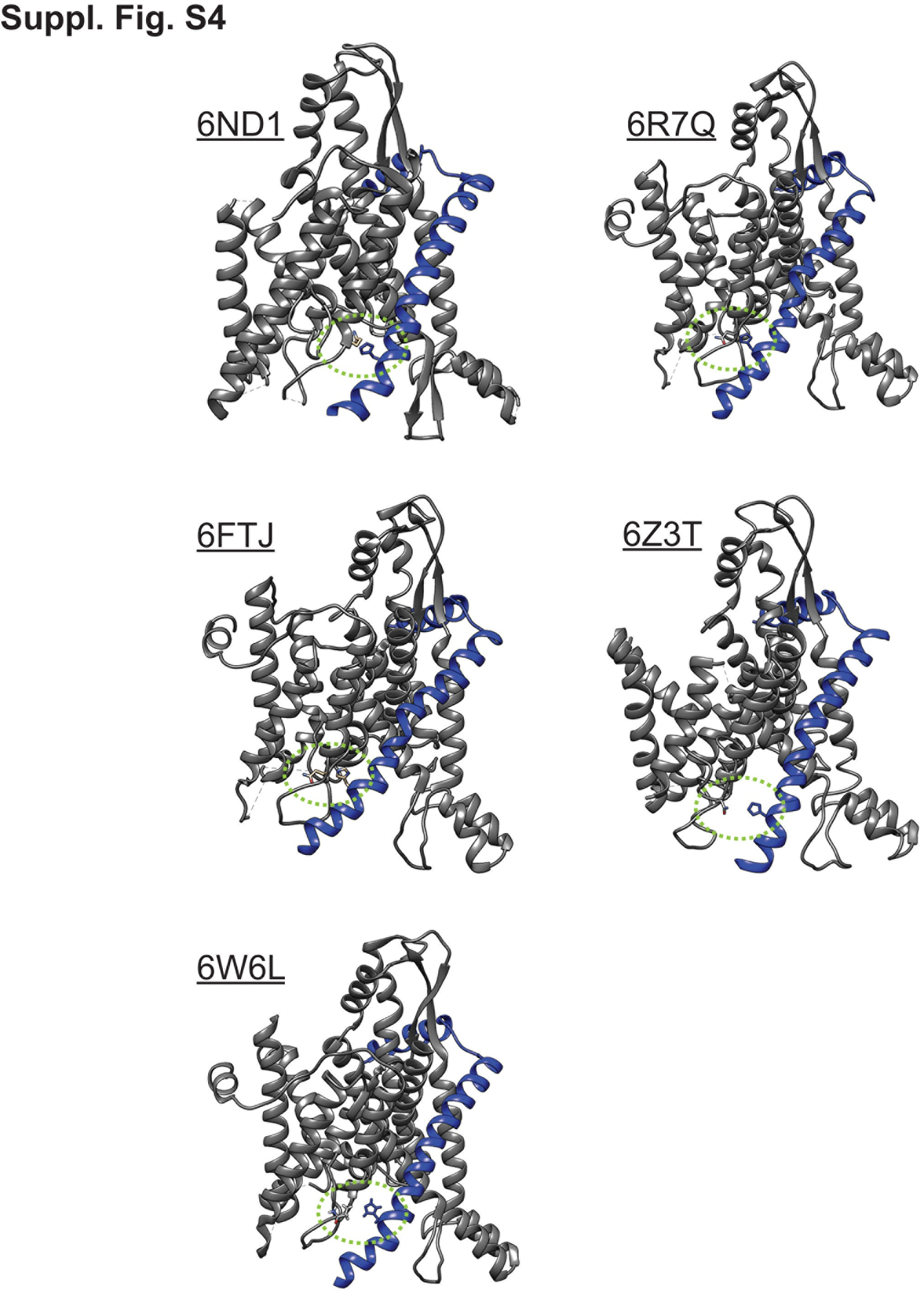

Supplement: S4 Fig — The position of Q47 in Sec61α relative to H58 in Sec61γ are indicated. (TIF) [file pgen.1009780.s004.tif]

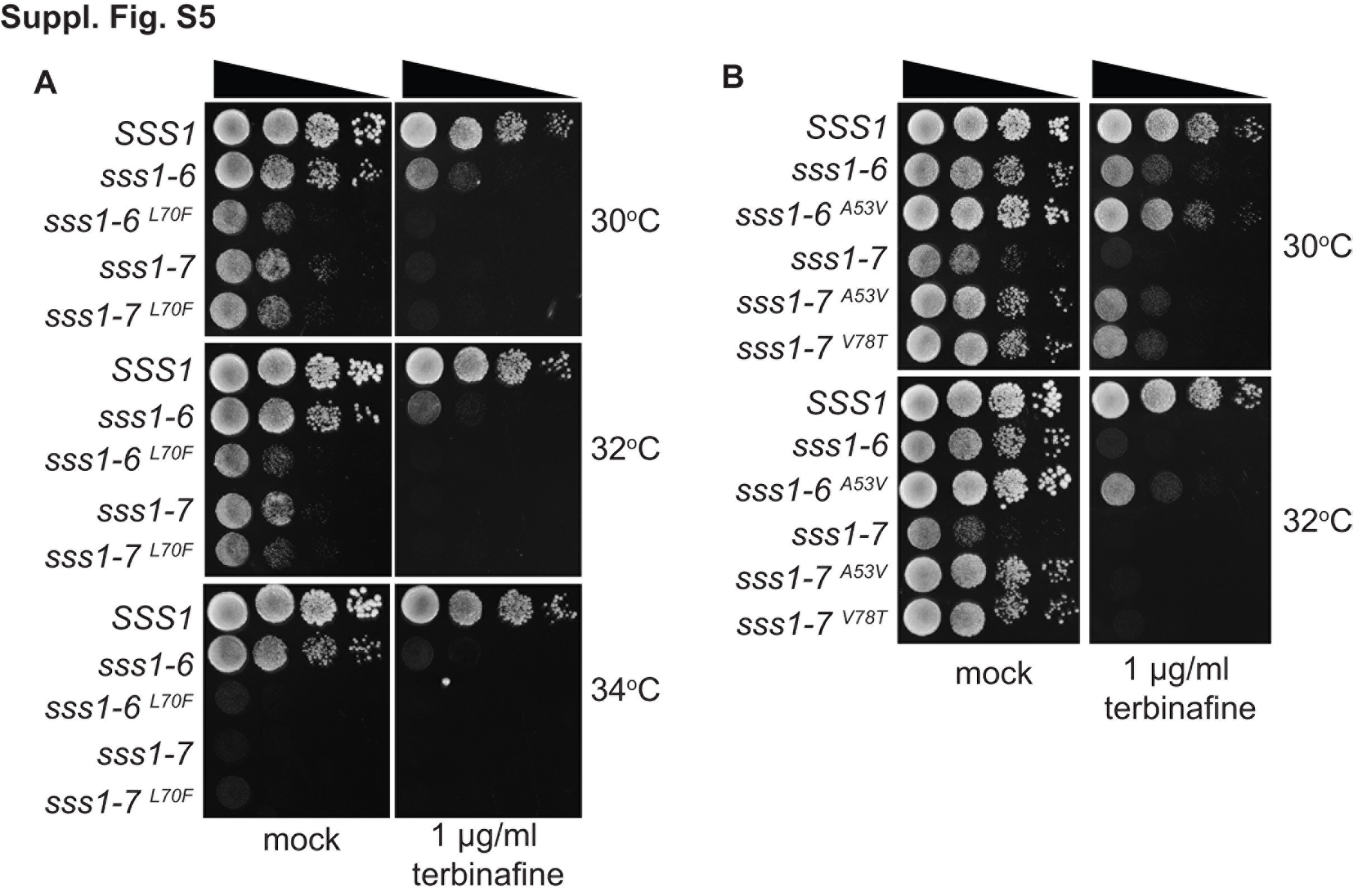

Supplement: S5 Fig — (A) Wildtype or cells expressing either SSS1P74A,I75A, SSS1L70F, P74A,I75A, SSS1H72K or SSS1L70F,H72K as the sole source of SSS1 were spotted on YPD agar or YPD agar containing 1 μg/mL terbinafine in a 10-fold dilution series and incubated at 30°C, 32°C or 34°C for 2 days. (B) Wildtype or cells expressing either SSS1P74A,I75A, SSS1A53V, P74A,I75A, SSS1H72K, SSS1A53V, H72K or SSS1H72K, V78T as the sole source of SSS1 were spotted on YPD agar or YPD agar containing 1 μg/mL terbinafine in a 10-fold dilution series and incubated at 30°C or 32°C for 2 days. (TIF) [file pgen.1009780.s005.tif]
