# Supplementary material for: Cancer associated mutations in Sec61γ alter the permeability of the ER translocase
Source: PLoS Genet. 2021 Aug 30;17(8):e1009780. doi: 10.1371/journal.pgen.1009780 (PMC8439465; doi:10.1371/journal.pgen.1009780)
Supplement: S1 Table — (PDF) [file pgen.1009780.s006.pdf]

S1 Table. Yeast strains used in this study.

Yeast	Genotype	Reference
BWY530	*MATα ade2- 1 ura3-1 his3-11,15 trp1-1 leu2-3,112 can1-100 sss1Δ::KanMX4* FKp53	Wilkinson et al. 2010 [1]
SSS1	*MATα ade2- 1 ura3-1 his3-11,15 trp1-1 leu2-3,112 can1-100 sss1Δ::KanMX4* pJKB2	Wilkinson et al. 2010 [1]
sss1-6	*MATα ade2- 1 ura3-1 his3-11,15 trp1-1 leu2-3,112 can1-100 sss1Δ::KanMX4* pJKB16	Witham et al., 2020 [2]
sss1-7	*MATα ade2- 1 ura3-1 his3-11,15 trp1-1 leu2-3,112 can1-100 sss1Δ::KanMX4* pCM205	Witham et al., 2020 [2]
sss1-8	*MATα ade2- 1 ura3-1 his3-11,15 trp1-1 leu2-3,112 can1-100 sss1Δ::KanMX4* pCW11	This study
sss1-KI	*MATα ade2- 1 ura3-1 his3-11,15 trp1-1 leu2-3,112 can1-100 sss1Δ::KanMX4* pCW12	This study
sss1-KE	*MATα ade2- 1 ura3-1 his3-11,15 trp1-1 leu2-3,112 can1-100 sss1Δ::KanMX4* pCW13	This study
sss1-AV	*MATα ade2- 1 ura3-1 his3-11,15 trp1-1 leu2-3,112 can1-100 sss1Δ::KanMX4* pCW14	This study
sss1-LF	*MATα ade2- 1 ura3-1 his3-11,15 trp1-1 leu2-3,112 can1-100 sss1Δ::KanMX4* pCW15	This study
sss1-VT	*MATα ade2- 1 ura3-1 his3-11,15 trp1-1 leu2-3,112 can1-100 sss1Δ::KanMX4* pCW16	This study
sss1-6 KI	*MATα ade2- 1 ura3-1 his3-11,15 trp1-1 leu2-3,112 can1-100 sss1Δ::KanMX4* pCW17	This study
sss1-6 KE	*MATα ade2- 1 ura3-1 his3-11,15 trp1-1 leu2-3,112 can1-100 sss1Δ::KanMX4* pCW18	This study
sss1-6 AV	*MATα ade2- 1 ura3-1 his3-11,15 trp1-1 leu2-3,112 can1-100 sss1Δ::KanMX4* pCW19	This study
sss1-6 LF	*MATα ade2- 1 ura3-1 his3-11,15 trp1-1 leu2-3,112 can1-100 sss1Δ::KanMX4* pCW20	This study
sss1-6 VT	*MATα ade2- 1 ura3-1 his3-11,15 trp1-1 leu2-3,112 can1-100 sss1Δ::KanMX4* pCW21	This study
sss1-7 KI	*MATα ade2- 1 ura3-1 his3-11,15 trp1-1 leu2-3,112 can1-100 sss1Δ::KanMX4* pCW22	This study

sss1-7 KE	*MATα ade2- 1 ura3-1 his3-11,15 trp1-1 leu2-3,112 can1-100 sss1Δ::KanMX4* pCW23	This study
sss1-7 AV	*MATα ade2- 1 ura3-1 his3-11,15 trp1-1 leu2-3,112 can1-100 sss1Δ::KanMX4* pCW24	This study
sss1-7 LF	*MATα ade2- 1 ura3-1 his3-11,15 trp1-1 leu2-3,112 can1-100 sss1Δ::KanMX4* pCW25	This study
sss1-7 VT	*MATα ade2- 1 ura3-1 his3-11,15 trp1-1 leu2-3,112 can1-100 sss1Δ::KanMX4* pCW26	This study
CWY46	*sss1-8* + YCp *SEC61* (pBW11)	This study
CWY47	*sss1-8* + YCp $SEC61^{N302K}$ (pCW4)	This study
CWY48	*sss1-8* + YCp $SEC61^{N302L}$ (pCW7)	This study
CWY49	*sss1-8* + YCp $SEC61^{Q48A}$ (pCW27)	This study
CWY50	*sss1-6 KE* + pRS315	This study
CWY51	*sss1-6 KE* + YCp *SEC61* (pBW11)	This study
CWY52	*sss1-6 KE* + YCp $SEC61^{N302L}$ (pCW7)	This study
CWY53	*sss1-6 KE* + YCp $SEC61^{N302K}$ (pCW4)	This study
CWY54	*sss1-7 KE* + pRS315	This study
CWY55	*sss1-7 KE* + YCp *SEC61* (pBW11)	This study
CWY56	*sss1-7 KE* + YCp $SEC61^{N302L}$ (pCW7)	This study
CWY57	*sss1-7 KE* + YCp $SEC61^{N302K}$ (pCW4)	This study
CWY58	*SSS1*+ YCp *SEC61* (pBW11) + pJT30	Witham et al., 2020 [2]
CWY59	*sss1-6* + YCp *SEC61* (pBW11) + pJT30	Witham et al., 2020 [2]
CWY60	*sss1-7* + YCp *SEC61* (pBW11) + pJT30	Witham et al., 2020 [2]
CWY61	*sss1-6 LF* + YCp *SEC61* (pBW11) + pJT30	This study
CWY62	*sss1-7 LF* + YCp *SEC61* (pBW11) + pJT30	This study
CWY63	*sss1-6 AV* + YCp *SEC61* (pBW11) + pJT30	This study
CWY64	*sss1-7 AV* + YCp *SEC61* (pBW11) + pJT30	This study
CWY65	*sss1-7 VT* + YCp *SEC61* (pBW11) + pJT30	This study
CWY66	*sss1-8* + YCp *SEC61* (pBW11) + pJT30	This study
CWY67	*sss1-8* + YCp $SEC61^{N302K}$ (pCW4) + pJT30	This study

CWY68	*sss1-8* + YCp *SEC61*^{N302L} (pCW7) + pJT30	This study
CWY69	*sss1-8* + YCp *SEC61*^{Q48A} (pCW27) + pJT30	This study
CWY70	*SSS1* + YCp *SEC61* (pBW11) + pCW10	Witham et al., 2020 [2]
CWY71	*sss1-6* + YCp *SEC61* (pBW11) + pCW10	Witham et al., 2020 [2]
CWY72	*sss1-7* + YCp *SEC61* (pBW11) + pCW10	Witham et al., 2020 [2]
CWY73	*sss1-6 LF* + YCp *SEC61* (pBW11) + pCW10	This study
CWY74	*sss1-7 LF* + YCp *SEC61* (pBW11) + pCW10	This study
CWY75	*sss1-6 AV* + YCp *SEC61* (pBW11) + pCW10	This study
CWY76	*sss1-7 AV* + YCp *SEC61* (pBW11) + pCW10	This study
CWY77	*sss1-7 VT* + YCp *SEC61* (pBW11) + pCW10	This study
CWY78	*sss1-8* + YCp *SEC61* (pBW11) + pCW10	This study
CWY79	*sss1-8* + YCp *SEC61*^{N302K} (pCW4) + pCW10	This study
CWY80	*sss1-8* + YCp *SEC61*^{N302L} (pCW7) + pCW10	This study
CWY81	*sss1-8* + YCp *SEC61*^{Q48A} (pCW27) + pCW10	This study

References

1. Wilkinson BM, Brownsword JK, Mousley CJ, Stirling CJ. Sss1p Is Required to Complete Protein Translocon Activation. J. Biol. Chem. 2010; 285(42):32671-7.

2. Witham CM, Dassanayake HG, Paxman AL, Stevens KLP, Baklous L, White PF, et al. The conserved C-terminus of Sss1p is required to maintain the endoplasmic reticulum permeability barrier. J. Biol. Chem. 2020; 295(7):2125-34.
