# Supplementary material for: Cancer associated mutations in Sec61γ alter the permeability of the ER translocase
Source: PLoS Genet. 2021 Aug 30;17(8):e1009780. doi: 10.1371/journal.pgen.1009780 (PMC8439465; doi:10.1371/journal.pgen.1009780)
Supplement: S2 Table — (PDF) [file pgen.1009780.s007.pdf]

S2 Table. Plasmids used in this study.

Plasmid	Description	Reference
pJT30	UPRE-LACZ reporter	Tyson and Stirling, 2000 [1]
pBW11	YCp *SEC61 LEU2*	Wilkinson et al., 1997 [2]
pDR195	YEp *URA3* containing the *PMA1* promoter and *CYC1* terminator to enable high level gene expression.	Rentsch et al. 1995 [3]
pJKB2	YCp *SSS1 HIS3*	Wilkinson et al., 2010. [4]
pJKB16	YCp $sss1^{P74A\ I75A}$ *HIS3*	Witham et al., 2020 [5]
pCM203	YCp *SEC61 SSS1 URA3*	Witham et al., 2020 [5]
pCM205	YCp $sss1^{H72K}$ *HIS3*	Witham et al., 2020 [5]
pCW4	YCp $SEC61^{N302K}$ *LEU2*	Witham et al., 2020 [5]
pCW7	YCp $SEC61^{N302L}$ *LEU2*	Witham et al., 2020 [5]
pCW10	YEp *HGT1 URA3*	Witham et al., 2020 [5]
pCW11	YCp $sss1^{H72R}$ *HIS3*	This study
pCW12	YCp $sss1^{K38I}$ *HIS3*	This study
pCW13	YCp $sss1^{K41E}$ *HIS3*	This study
pCW14	YCp $sss1^{A53V}$ *HIS3*	This study
pCW15	YCp $sss1^{L70F}$ *HIS3*	This study
pCW16	YCp $sss1^{V78T}$ *HIS3*	This study
pCW17	YCp $sss1^{K38I\ P74A\ I75A}$ *HIS3*	This study
pCW18	YCp $sss1^{K41E\ P74A\ I75A}$ *HIS3*	This study
pCW19	YCp $sss1^{A53V\ P74A\ I75A}$ *HIS3*	This study
pCW20	YCp $sss1^{L70F\ P74A\ I75A}$ *HIS3*	This study
pCW21	YCp $sss1^{V78T\ P74A\ I75A}$ *HIS3*	This study
pCW22	YCp $sss1^{K38I\ H72K}$ *HIS3*	This study

pCW23	YCp *sss1*$^{K41E\ H72K}$ *HIS3*	This study
pCW24	YCp *sss1*$^{A53V\ H72K}$ *HIS3*	This study
pCW25	YCp *sss1*$^{L70F\ H72K}$ *HIS3*	This study
pCW26	YCp *sss1*$^{V78T\ H72K}$ *HIS3*	This study
pCW27	YCp *SEC61*^{Q48A} *LEU2*	This study

References

1. Tyson JR, Stirling CJ. LHS1 and SIL1 provide a lumenal function that is essential for protein translocation into the endoplasmic reticulum. EMBO J. 2000; 19(23):6440-52.

2. Wilkinson BM, Esnault Y, Craven RA, Skiba F, Fieschi J, K'Epes F, et al. Molecular architecture of the ER translocase probed by chemical crosslinking of Sss1p to complementary fragments of Sec61p. EMBO J. 1997; 16(15):4549-59.

3. D. Rentsch et al., NTR1 encodes a high affinity oligopeptide transporter in Arabidopsis. FEBS letters 370, 264-268 (1995).

4. Wilkinson BM, Brownsword JK, Mousley CJ, Stirling CJ. Sss1p Is Required to Complete Protein Translocon Activation. J. Biol. Chem. 2010; 285(42):32671-7.

5. Witham CM, Dassanayake HG, Paxman AL, Stevens KLP, Baklous L, White PF, et al. The conserved C-terminus of Sss1p is required to maintain the endoplasmic reticulum permeability barrier. J. Biol. Chem. 2020; 295(7):2125-34.
