# Supplementary material for: Cancer associated mutations in Sec61γ alter the permeability of the ER translocase
Source: PLoS Genet. 2021 Aug 30;17(8):e1009780. doi: 10.1371/journal.pgen.1009780 (PMC8439465; doi:10.1371/journal.pgen.1009780)
Supplement: S3 Table — (PDF) [file pgen.1009780.s008.pdf]

S3 Table. Oligonucleotides used in this study.

Name	Sequence
SSS1 P74A I75A_F	GATTCATATTgCAgcCAGATACGTTATTGT
SSS1 P74A I75A_R	GTATCTGgcTGcAATATGAATCAACTTGAT
SSS1 H72K_F	AAGTTGATTaAaATTCCAATCAGATACGTTATTG
SSS1 H72K_R	TGATTGGAATtTtAATCAACTTGATGGCGTAAC
SSS1 K38I_F	AATTCTTGGCCAttTGTAAGAAACCTGATT
SSS1 K38I _R	TTCTTACAaaTGGCCAAGAATTGAGTACCT
SSS1 K41E_F	CAAGTGTAAGgAACCTGATTTGAAGGAATA
SSS1 K41E_R	CAAATCAGGTTcCTTACACTTGGCCAAGAA
SSS1 A53V_F	GATTGTCAAGGtTGTTGGTATTGGTTTTAT
SSS1 A53V_R	AATACCAACAaCCTTGACAATCTTGGTGTA
SSS1 L70F_F	CATCAAGTTtATTCATATTCCAATCAGATACG
SSS1 L70F_R	GCTGCAATATGAATaAACTTGATGGCGTAACC
SSS1 H72R_F	CATCAAGTTGATTagaATTCCAATCAGATACG
SSS1 H72R_R	ATTGGAATtctAATCAACTTGATGGCGTAACC
SSS1 V78T_F	CAGATACacTATTGTTTAAAAGAGATAAAAG
SSS1 V78T_R	TCTTTTAAACAATAgtGTATCTGATTGGAAT
SSS1 L70F P74A I75A_F	CATCAAGTTtATTCATATTGCAGCCAGATACG
SSS1 L70F P74A I75A_R	ATTGGAATATGAATaAACTTGATGGCGTAACC
SSS1 L70F H72K_F	CATCAAGTTtATTaAaATTCCAATCAGATACG
SSS1 L70F H72K_R	ATTGGAATtTtAATaAACTTGATGGCGTAACC
SSS1 V78T P74A I75A_R	TCTTTTAAACAATAgtGTATCTGGCTGGAAT
HGT1_qpcr_F	CCCAATTGGTAGGATACTGG
HGT1_qpcr_R	GTAAGACCTGCAGCACCATAAC
PMR1_qpcr_F	GGCAACCAAGATTCTCAACC
PMR1_qpcr_R	GCCATAGAATTTGCGCACTC
ACT1_qpcr_F	GCCTTCTACGTTTCCATCCA
ACT1_qpcr_R	GGCCAAATCGATTCTCAAAA
