# Supplementary material for: Cancer associated mutations in Sec61γ alter the permeability of the ER translocase
Source: PLoS Genet. 2021 Aug 30;17(8):e1009780. doi: 10.1371/journal.pgen.1009780 (PMC8439465; doi:10.1371/journal.pgen.1009780)
Supplement: S4 Table — (PDF) [file pgen.1009780.s009.pdf]

**S4 Table. Antibodies used in this study.**

| Antibody | Dilution | Reference |
|---|---|---|
| Anti-Sec61p, rabbit polyclonal | 1:10,000 | Stirling et al., 1992 [1] |
| Anti-Sss1p, sheep polyclonal | 1:5000 | Wilkinson et al., 2010 [2] |
| Anti-Sec63p, sheep polyclonal | 1:10,000 | Young et al., 2001 [3] |
| Anti-DPAP B, sheep polyclonal | 1:5000 | Tyson and Stirling 2000 [4] |
| Anti-Kar2p, sheep polyclonal | 1:10,000 | Tyson and Stirling 2000 [4] |
| Anti-Prepro alpha factor | 1:10,000 | Young et al., 2001 [3] |
| HRP conjugated anti-sheep | 1:20,000 | N/A |
| HRP conjugated anti-rabbit | 1:20,000 | N/A |

**References**


1. C. J. Stirling, J. Rothblatt, M. Hosobuchi, R. Deshaies, R. Schekman, Protein translocation mutants defective in the insertion of integral membrane proteins into the endoplasmic reticulum. Mol. Biol. Cell 3, 129-142 (1992).

2. Wilkinson BM, Brownsword JK, Mousley CJ, Stirling CJ. Sss1p Is Required to Complete Protein Translocon Activation. J. Biol. Chem. 2010; 285(42):32671-7.

3. B. P. Young, R. A. Craven, P. J. Reid, M. Willer, C. J. Stirling, Sec63p and Kar2p are required for the translocation of SRP-dependent precursors into the yeast endoplasmic reticulum in vivo. EMBO J. 20, 262-271 (2001).

4. Tyson JR, Stirling CJ. LHS1 and SIL1 provide a lumenal function that is essential for protein translocation into the endoplasmic reticulum. EMBO J. 2000; 19(23):6440-52.
