# Supplementary material for: Cancer associated mutations in Sec61γ alter the permeability of the ER translocase
Source: PLoS Genet. 2021 Aug 30;17(8):e1009780. doi: 10.1371/journal.pgen.1009780 (PMC8439465; doi:10.1371/journal.pgen.1009780)
Supplement: S1 Methods — (PDF) [file pgen.1009780.s010.pdf]

Supplemental materials and methods

Invertase secretion

Cells were grown in YPD (2% glucose) at 30°C, cultures were split, and cells incubated at 30°C or 37°C for 1h in YPD (2% glucose). Cells were then pelleted (2000g), washed twice with pre-warmed low glucose (0.1% glucose) YPD, resuspended in the low glucose YPD medium, and incubated as before for 1.5h. To halt trafficking, samples were adjusted to 10mM NaN_3, and incubated on ice. The samples were washed 3X with 500µl ice-cold 10mM NaN_3 and re-suspended in 500µl of the same. The samples were split into 10mM NaN_3 buffers ± 0.2 % Triton X-100 (final) with the Triton-solubiliaed fractions also being subjected to one cycle of freeze-thaw to generate the permeabilised cell fraction. The partner non-permeabilised and permeabilised samples were used to determine extracellular and total invertase activities, respectively. The pool of secreted invertase is expressed as a fraction.

DSS cross-linking

Yeast microsomes were prepared according to Rothblatt and Meyer, 1986 [1]. Microsomes were incubated at 30°C for 30 min in the presence of either 1 mM DSS or equivalent volume of DMSO. The addition of 10 mM lysine and 10 mM Tris (final concentration) supplied excess amine groups to quench the reaction. Crosslinking was assessed through subsequent SDS PAGE, transfer and immunoblot.

Blue Native PAGE Analysis

BN-PAGE was performed according to Jermy et al., 2006 [2]. Two A_{280}nm units of microsomes were harvested by centrifugation at 10,000 × g, resuspended in 100 µl of S-buffer (20 mM Tris-HCl, pH 7.6, 250 mM NaCl, 2 mM dithiothreitol, 5 mM MgOAc, 2% digitonin, 12% glycerol, 1% (v/v) protease inhibitor mixture (Sigma)) and then incubated on ice for 30

min. Unsolubilised material was removed by centrifugation at 10,000 × g, and then ribosomes were removed by centrifugation for 60 min at 400,000 × g. The supernatant was then diluted to 180 µl with S-buffer without NaCl and digitonin, followed by the addition of 20 µl of 10× sample buffer (5% Coomassie Brilliant Blue G250, 500 mM 6-aminocaproic acid, 100 mM Bistris-HCl, pH 7.0). 0.8 A_{280}nm unit aliquots were loaded onto a 6–16% polyacrylamide gradient gel (buffered with 500 mM 6-aminocaproic acid, 50 mM Tris-HCl, pH 7.0). The samples were run at 200 V for 18 h with Coomassie-containing cathode buffer (50 mM Tricine, pH 7.0, 15 mM Bistris-HCl, pH 7.0, 0.02% Coomassie Brilliant Blue G250) and then for a further 3 h at 500 V in buffer lacking Coomassie (50 mM Tricine, pH 7.0, 15 mM Bistris-HCl, pH 7.0). Anode buffer (50 mM Bistris-HCl, pH 7.0) was constant throughout. The samples were then transferred to polyvinylidene difluoride membrane and analysed by Western blotting.

Reverse transcription (RT)-PCR

Total RNA was isolated from cells using the FavorPrepTM blood cultured cell total RNA mini Kit and 1µg was used to template reverse transcription using WarmStart RTx Reverse Transcriptase to generate cDNA (20µl final volume). H_2O was used in no RT controls. To analyse *HGT1*, *PMR1* or *ACT1* expression, PCR was performed using 1µl of cDNA fraction as template and specific oligonucleotides as primers. Products were quantified with Image J software.

References

1. J. A. Rothblatt, D. I. Meyer, Secretion in yeast: translocation and glycosylation of prepro-alpha-factor in vitro can occur via an ATP-dependent post-translational mechanism. EMBO J. 5, 1031-1036 (1986).

2. Jermy AJ, Willer M, Davis E, Wilkinson BM, Stirling CJ. The Brl domain in Sec63p is required for assembly of functional endoplasmic reticulum translocons. J. Biol. Chem. 2006; 281(12):7899-906.
